# Development of DuoChol, a Thermostable Inactivated Whole-Cell/B-Subunit Oral Cholera Vaccine in Enteric Capsule

**DOI:** 10.3390/vaccines14070573

**Published:** 2026-06-29

**Authors:** Manuela Terrinoni, Michael R. Lebens, Stefan L. Nordqvist, Frida Nilsson, Madeleine Löfstrand, Julia Lynch, Jan Holmgren

**Affiliations:** 1 Department of Microbiology and Immunology, Gothenburg University Vaccine Research Institute (GUVAX), Institute of Biomedicine, Sahlgrenska Academy at University of Gothenburg, Box 435, 405 30 Gothenburg, Sweden; 2 International Vaccine Institute (IVI), Seoul 08826, Republic of Korea; 3 Gotovax AB, 426 74 Gothenburg, Sweden

**Keywords:** cholera, vaccine, *Vibrio cholerae*, mucosal immunity, cholera toxin, enteric capsule, gut immunity

## Abstract

**Background/Objectives**: Cholera remains an important global health problem. Inactivated oral cholera vaccines (OCVs) are essential in the WHO/GTFCC (World Health Organization/Global Task Force on Cholera Control) strategy to end cholera by 2030; however, global supply is insufficient, they require partial cold-chain storage, and their formulation and antigen contents leave room for improvement. We describe here the development and preclinical evaluation of DuoChol OCV, a next-generation thermostable oral vaccine designed to address these gaps. **Methods**: DuoChol is a lyophilized dry-powder formulation in enteric capsules containing formalin-inactivated *Vibrio cholerae* O1 El Tor Ogawa and Inaba isogenic bacteria, recombinant cholera toxin B subunit (rCTB), and sucrose as stabilizer. Methods describe the construction of the novel vaccine strains, processes for the preparation and characterization of vaccine components, and the final dry formulation in enteric capsules, and in vitro and in vivo vaccine stability analyses. **Results**: The newly engineered vaccine strains, together with a high-yield mixed-mode chromatography process for rCTB purification, enabled efficient and cost-effective vaccine production. Stability studies demonstrated complete preservation of O1 LPS and rCTB antigens for at least 21 months across temperatures of 4–40 °C. Moreover, regardless of storage duration or temperature, oral immunization of mice with DuoChol elicited strong serum and mucosal antibacterial and antitoxin responses that were similar to those induced by the licensed Dukoral^®^ OCV. **Conclusions**: Its heat stability, practical enteric capsule formulation, and potential for improved efficacy compared to inactivated whole-cell only OCVs support positioning DuoChol as a promising next-generation OCV, suitable for national cholera control programs and particularly advantageous for outbreak response, where rapid deployment and early, robust protection are essential.

## 1. Introduction

Cholera remains a major global health threat, particularly in regions lacking safe water and sanitation. Infection may follow ingestion of food or water contaminated with *Vibrio cholerae* and cause acute watery diarrhea that can rapidly lead to dehydration and death without treatment. Nearly all cases in the past two decades have been caused by *V. cholerae* O1, serotypes Inaba and Ogawa, of the El Tor biotype. Despite being preventable and treatable, cholera causes an estimated 4 million cases and 140,000 deaths annually [1,2,3]. Since 2022, large epidemics across the Middle East, Sub-Saharan Africa, and Southeast Asia, driven by climate change, extreme weather, conflicts, and displacement prompted the WHO to declare cholera a Grade 3 emergency [4,5,6,7,8,9,10,11].

While cholera control traditionally relies on efforts to improve water, sanitation, and hygiene (WASH) conditions, the limitations in infrastructure in many endemic regions have made oral cholera vaccines (OCVs) an essential additional tool. Vaccine protection depends on mucosal immunity, particularly SIgA responses to lipopolysaccharide (LPS) O antigen and cholera toxin (CT) [12,13,14,15,16,17]. Early parenteral vaccines gave only modest and short-lived protection and were reactogenic, leading to their replacement by OCVs that provide multi-year protection and confer additional herd effects [18,19,20,21,22,23]. WHO currently recommends several multicomponent, inactivated whole-cell OCVs—Dukoral^®^, Shanchol™, and Euvichol-Plus™—for both preventive use and outbreak response. Since 2013, more than 250 million doses have been deployed through the global OCV stockpile, though demand continues to exceed supply, which was worsened by the discontinuation of Shanchol™ in 2023.

OCVs have well-documented preventive impact, with meta-analyses showing two-dose efficacy of 55% and effectiveness of 69% at 12 months, declining to 44% and 47% by 48 months [24,25,26,27,28]. Supply constraints have recently forced single-dose campaigns, raising concerns about reduced protection, especially in non-endemic settings, among young children, and high-transmission outbreaks [29,30,31,32]. New, simplified, and cheaper to produce OCVs aim to address these gaps. Euvichol-S™, a two-component formulation comprising formalin-killed classical biotype Ogawa and El Tor biotype Inaba bacteria, and Hillchol™, a single-component Hikojima-based vaccine comprising formalin-killed El Tor bacteria co-expressing the Inaba and Ogawa serotype antigens, were recently licensed [33,34].

Further improvements in formulation and antigen composition may increase both the protective impact and practicality of OCV. Current OCVs require cold-chain storage, and all except Dukoral^®^ lack the CTB-subunit protein (CTB), which enhances short-term protection against cholera and provides cross-protection against enterotoxigenic *E. coli* diarrhea [35,36] but was omitted from subsequent OCVs due to cost and the requirement for co-administration with a buffer. We recently demonstrated that a lyophilized formulation containing formalin-killed *V. cholerae* O1 bacteria, recombinant CTB (rCTB), and sucrose was stable for over two years at 25 °C and for at least eight months at 40 °C [37].

Building on this, as described in this study, we have developed DuoChol, a dry-powder OCV in an enteric capsule containing lyophilized formalin-inactivated, newly generated isogenic *V. cholerae* O1 Ogawa and Inaba strains together with rCTB and sucrose as a stabilizer. Stability studies show preserved O1 LPS and rCTB antigens for at least 21 months at 4–40 °C as well as robust systemic and mucosal immunogenicity in mice, similar to that of side-by-side tested Dukoral OCV, after oral immunization.

## 2. Materials and Methods

### 2.1. Bacterial Strains and Seed Lots

New *V. cholerae* O1 vaccine strains were generated: two El Tor strains, MS1955 serotype Inaba and MS1987 serotype Ogawa, that are isogenic except for the serotype-determining *wbeT* gene, for the preparation of the DuoChol whole-cell components, and a classical biotype strain, MMS1692, engineered to express high levels of rCTB constitutively, for production of the rCTB vaccine component. The construction of these strains is detailed in the Supplementary File, Section A.

Master and working seeds of a lot of these vaccine strains were prepared in LB broth supplemented with glycerol (17% final concentration) and stored at −70 °C as described [38].

The vaccine strains, as well as the master and working seed lots, were analyzed to confirm their correct genotype and phenotype. The seed lots were analyzed by whole genome, next-generation sequencing (NGS) through outsourcing to Eurofins Genomics (Ebersberg, Germany) and further genotypic verification was performed using PCR as described in Supplementary File, Section B. Phenotypic characterization included evaluation of cell morphology through microscopy, colony-forming homogeneity on LB agar plates, and confirmation of biotype through growth in presence of polymyxin and of serotype through slide agglutination in specific antisera.

### 2.2. Media

LB agar plates were used for the culture of bacteria on solid medium, and LB broth for small-scale liquid cultures. For fermenter cultures of MS1955 and MS1987 strains “*Vibrio cholerae* growth medium (VCG)” was used [38]. For fermentation of the MMS1692 strain a Modified Syncase/Yeast extract (MS/YE) medium was used which contains per liter: Casamino acids, 20 g; Sucrose, 2.5 g; Na_2_HPO_4_2H_2_O, 6.27 g; K_2_HPO_4_, 5 g; NH_4_Cl, 1.18 g; Na_2_SO_4_, 0.089 g; MgCl_2_6H_2_O, 0.0042 g; MnCl_2_4H_2_O, 0.0004; FeCl_3_6H_2_O, 0.0005; Yeast Extract, 15 g. Added Feeding medium (MSF) contained per liter: 400 mM (72.5 g) Glucose in MS/YE.

All chemicals for media preparation were from Sigma Aldrich, except casamino acid and yeast extract, which were from Becton and Dickinson (BD).

### 2.3. Bacterial Cultures

Bacteria were grown at 37 °C unless otherwise indicated. Small-volume liquid shake cultures were undertaken in rotary shakers (180 rpm). Fermentations were done in a 3 Liter fermenter (Infors, Bottmingen, Switzerland).

#### 2.3.1. Fermentation of MS1955 and MS1987

The previously described conditions were used [38]. The culture was stopped when OD_600nm_ had reached between 25 and 30 (5–6 h) or 40–50 (8–9 h) and transferred into centrifugation bottles that were centrifuged at 10,000× *g* for 25 min at 4 °C. The supernatants were discarded, and the cell pellets resuspended in sterile phosphate-buffered saline (PBS), pooled, and adjusted/diluted with PBS to OD_600nm_ = 20 and then stored overnight at 4 °C for subsequent formalin inactivation as described below.

Samples taken when the fermentation was stopped, as well as from the centrifuged cell suspension were examined for *V. cholerae*-consistent morphology by phase contrast light microscopy, correct serotype by antibody slide agglutination, *V. cholerae*-consistent colonies of homogenous appearance by culture at 37 °C on LB agar plates, and content of *V. cholerae* O1 LPS antigen by Inhibition-ELISA (for description of assays see Section “Analytical Methods” below).

#### 2.3.2. Fermentation of MMS1692

A 2 L Erlenmeyer flask containing 250 mL of MS/YE medium was inoculated with MMS1692 from a working cell bank vial and incubated at 37 °C with shaking at 180 rpm until the culture OD_600nm_ reached 0.8–1.5 (approximately 5 h). This culture was used to inoculate 2.5 L of sterile pre-warmed MS/YE medium in the fermenter connected to a controlling unit. The pH was maintained at 7.2, the temperature at 37 °C, aeration at 2 reactor volumes/min, pO_2_ above 35% (using air and pure oxygen), and stirring at 800–1000 rpm. Feeding with MSF was initiated once the OD_600nm_ of the culture reached 2.0; the feed was given at a rate of 0.3–1.5 mL/min. Antifoam 204 (Sigma-Aldrich, St. Louis, MO, USA), diluted to 10% in water, was added as required to control foaming. The culture was stopped after 48 h, and the OD_600nm_ had reached between 10 and 12, and was transferred into centrifugation bottles that were centrifuged at 10,000× *g* for 25 min at 4 °C. The cell pellet was discarded, and the supernatant was saved and tested for its rCTB content by GM1-ELISA (see Section “Analytical Methods”). The supernatant was then processed for further purification of the rCTB as described below in Section 2.5.

### 2.4. Formalin Inactivation of Bacteria

Our previous work [37,38] showed that treatment of El Tor (or classical) *V. cholerae* O1 bacterial cultures with formalin at a concentration corresponding to 0.2 M formaldehyde (FA) at 22 ± 2 °C for 16–20 h resulted in complete bacterial killing with preserved bacterial morphology.

The same conditions were therefore used for the inactivation of the MS1955 and MS1987 fermenter-cultured bacteria. The inactivated bacteria were then centrifuged at 10,000× *g* for 25 min at 4 °C, the supernatant discarded, and the cell pellet resuspended in sterile PBS. This was repeated 4 times, whereafter the suspension was again centrifuged (10,000× *g* for 25 min), and the cell pellet was then resuspended in prewarmed (37 °C) 200 mM Tris-HCl/PBS pH 7.5 buffer and incubated at room temperature (RT) for 1 h. The suspension was again centrifuged (10,000× *g* for 25 min) and resuspended in PBS, a process repeated 4 times, and then adjusted with PBS to OD_600nm_ = 100 and stored in air-tight sterile glass bottles at 4 °C. A representative such as “Drug substance” (DS) preparation of each strain is referred to as “MS1955 Inaba DS” and “MS1987 Ogawa DS”, respectively.

Confirmation of complete inactivation was assessed as described [38] and levels of residual formaldehyde (with the extensive washing used <10 µg/mL) were determined with the Purpald method [39]. Each preparation was also examined for cell morphological appearance by light microscopy, bacterial serotype by slide agglutination, and content of O1 LPS antigen by Inhibition-ELISA.

### 2.5. Purification of rCTB

A novel mixed-mode chromatography (MMC) method for high-yield purification of rCTB was introduced and compared with the previously used Hexametaphosphate precipitation (HMP) and ion exchange chromatography method [40] as well as with a combination of HMP followed by heating at 65 °C [41] but in our hands, with the heating step performed after instead of before HMP [37].

#### 2.5.1. The MMC Method

After fermentation, the centrifuged MMS1692 culture supernatant was centrifuged once more at 10,000× *g* for 25 min, concentrated ten-fold using ultrafiltration (Amicon stirred cell, 400 mL capacity; 5 kDa cut-off membrane, Merck-Millipore, Darmstadt, Germany), and then buffer-exchanged into Binding Buffer (BB: 50 mM NaCl, 20 mM phosphate buffer, pH 7.4) by dialysis or diafiltration and filtered through a Millipore 0.2 µm membrane. A small sample of the concentrated material was analyzed by Nanodrop at 280 nm to estimate protein concentration.

Purification of rCTB was then performed on a 100 mL HiScreen™ Capto™ adhere column (Cytiva, Uppsala, Sweden) connected to an NGC FPLC system (Bio-Rad, Hercules, CA, USA) and equilibrated with 5 column volumes (CVs) of BB at a flow rate of 5 mL/min to give a stable baseline.

Adjusted to the protein content, approximately 300 mL of the crude rCTB material was then loaded onto the equilibrated column at 5 mL/min. After loading, the column was washed with 15 CVs of BB at 15 mL/min. 

Bound proteins were eluted using 10 CVs of a 70:30 mixture of Elution Buffer (EB: 400 mM NaCl, 20 mM citrate-phosphate buffer, pH 3) and BB, giving a final elution buffer composition of 295 mM NaCl in 20 mM phosphate buffer, pH 4. The elution flow rate was 10 mL/min, and the eluted material was monitored by UV spectrophotometry at A_280nm_.

Eluted fractions were collected into vials pre-loaded with 1/5 volume of 50 mM Trizma Base (pH 9) to ensure rapid pH neutralization. A major protein peak was obtained and shown by SDS-PAGE to contain highly purified rCTB. Collection of purified rCTB started when the UV signal had increased to 300 mAU and was stopped when it had returned to 300 mAU. The total collected rCTB volume was approximately one CV (80–100 mL). A final 100%EB elution step was applied to complete the gradient.

The rCTB fractions were pooled, buffer-exchanged into PBS by diafiltration or dialysis, examined for their concentration with a nanodrop method, concentrated to 10–20 mg/mL by ultrafiltration (Amicon 5 kDa membrane, Merck Millipore Ltd., Cork, Ireland), and sterile-filtered through a 0.2 µm filter (Millipore, Merck Millipore Ltd., Cork, Ireland). A representative preparation was referred to as “rCTB DS.”

#### 2.5.2. Characterization of Purified rCTB

Purified rCTB prepared by the different methods was analyzed by several methods: (i) SDS-PAGE/Densitometry for purity; (ii) size exclusion chromatography (SEC) on a preparative Superdex200 26/60 column (Cytiva, Uppsala, Sweden) [37] for oligomeric migration profile; and (iii) GM1-ELISA [42] for concentration as well as receptor- and antibody-binding activities (and by comparison with the starting supernatant also for recovery yield).

### 2.6. Lyophilization and Capsule Filling

**Figure 1:**
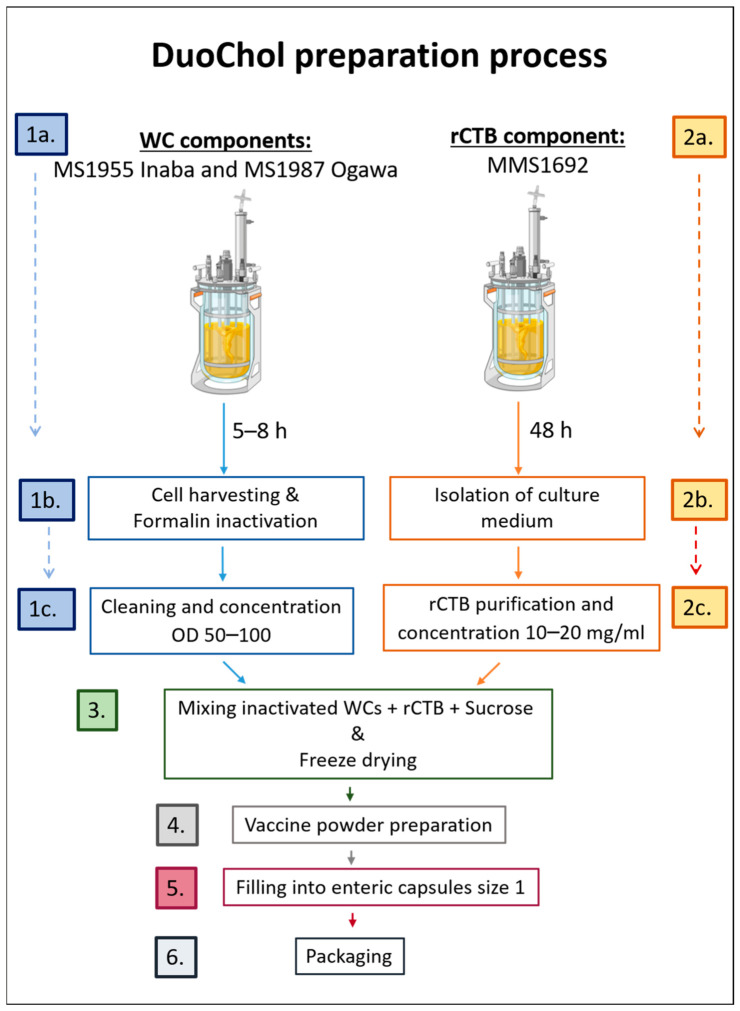
Process description of the DuoChol inactivated whole-cell (WC) and rCTB OCV. 1a. Fermentation of isogenic strains MS1955 and MS1987 (to OD_600nm_ 40–50); 1b. Harvesting and formalin inactivation of bacterial cells; 1c. Washing and concentration of inactivated cells (Inaba and Ogawa Drug Substance [DS], respectively); 2a. Fermentation of MMS1692 for rCTB production; 2b. Harvesting of culture medium; 2c. Purification and concentration of rCTB (rCTB DS); 3. Mixing Inaba and Ogawa Whole-cell and rCTB DSs with sucrose and freeze–drying; 4. Vaccine powder preparation by milling with a mortar and pestle and particle sieving; 5. Powder filling into size 1 enteric capsules (DuoChol OCV “Drug Product”, DP); 6. Packaging for storage and analysis.

A mixture of formalin-inactivated whole-cell components, purified rCTB, and sucrose was lyophilized for subsequent preparation of freeze-dried powder to be used for filling in capsules (see Figure 1, steps 3–6). These steps were performed at RISE (Research Institute of Sweden, Unit of Drug formulation, Södertälje). The freeze-drying (FD) procedure, earlier described [37], was used with the small modifications of using a higher concentration of sucrose (60 mg/mL) and scaling up to provide sufficient dried vaccine powder for filling 150–200 capsules. The liquid mixture used for freeze-drying contained per ml the formalin-inactivated Inaba and Ogawa components in an amount providing 0.75 mg O1 LPS antigen in each, 1 mg rCTB, 75 mg sucrose, and 5 mg buffer salts. With no loss in the lyophilization and further steps, this was calculated to provide ca 150 mg dry powder per ml liquid mixture, with the bacterial components accounting for a dry weight of 35 mg each. The inactivated bacterial suspension bulks were carefully shaken to provide an even suspension for the mixing, and the mixture was then again shaken vigorously and poured into a circular lyophilization tray with a 23 cm diameter, so that a volume of 200 mL would give a fill height of 0.48 cm. The tray was placed on the shelf of an Epsilon 2–4 LSCplus (Martin Christ GmbH, Osterode am Harz, Germany) freeze-dryer. The material was frozen at −45 °C for approximately 4 h and then subjected to a first drying cycle for 36 h at 0.0200 mbar at −30 °C, which was followed by warming the material to +20 °C and a second drying cycle for 1 h at +20 °C at 0.005 mbar.

The lyophilized “cake” was crushed and treated with a pestle and mortar to generate a free-flowing powder. The powder was transferred to a bottle that was filled with nitrogen gas to prevent moisture uptake. The whole procedure was conducted in a glove box with controlled low relative humidity. The powder was used to fill 150 size 1 enteric capsules (Capsugel^®^ Enprotect ^®^, Lonza, Basel, Switzerland). Capsules were packaged in an airtight flask together with desiccating powder; the remaining powder was stored in the presence of desiccating powder at 4 °C for reference purposes.

A prototype lot of such DuoChol vaccine capsules (“Drug Product”, DP) was manufactured at RISE using the Inaba DS, Ogawa DS, and rCTB DS; the residual moisture in the final powder used for capsule filling was 0.9% (*w*/*w*). These capsules were sent to us and used for further characterization, in vitro stability, and in vivo immunogenicity testing as described below.

### 2.7. In Vitro Stability Assays

Freshly prepared prototype DP vaccine capsules received from RISE, as well as lyophilized powder from the same lot, were analyzed (time 0, “t0”) as described below. Remaining capsules were distributed in airtight glass bottles with desiccating powder for storage at 4(±2) °C, 25(±2) °C, or 40(±2) °C; the reference powder was stored in the same way at 4(±2) °C. At 2 months (“t2”), 4 months (“t4”), 6 months (“t6”), and finally at 21 months (“t21”), three random capsules from each storage temperature were analyzed together with the reference powder stored at 4 °C.

Capsules were inspected to be intact, whereafter they were cut open, and the powder content was carefully weighed and visually inspected for any change in appearance and placed in a plastic vial. Milli-Q water was added to give a powder content concentration of 100 mg/mL. After careful mixing, the material was then analyzed side-by-side with the reference powder suspension for: (i) Optical density (OD_600nm_) [37]; (ii) O1 LPS antigen content by inhibition-ELISA [43]; (iii) rCTB antigen content by GM1-ELISA [37,42]; and (iv) Bacterial morphology and aggregation by light microscopy with the analytic methods described below.

### 2.8. Immunogenicity Studies

Immunogenicity of DP capsule vaccine after storage at different temperatures for 5 and 21 months was assessed and compared with that of cold-chain stored Dukoral OCV side-by-side by measuring serum and fecal (intestinal-mucosal) antibody responses in mice after oral/intragastric immunizations. Studies were performed in C57/BL6-Balb/c F1 hybrid mice (ENVIGO, Horst, The Netherlands); F1 hybrid mice were used to reduce the Th1 and Th2 immune response bias, respectively, of the parent mouse strains [44,45,46,47,48,49]. DuoChol vaccine capsules that had been stored at 4 °C, 25 °C, or 40 °C for 5 or 21 months were dissolved in PBS, and after mixing of an appropriate amount of dissolved vaccine with a sodium bicarbonate buffer, 1/20th of a human dose was given intragastrically in 150 µL 3% sodium carbonate solution to each of seven 6–8 week old female mice per test group; an administration that was repeated twice at 2-week intervals. For comparison, another group of 7 mice was immunized in the same way with a 1/20th human dose of cold-chain stored Dukoral OCV. This dose was chosen as pretests with both Duochol and licensed Dukoral, Shanchol, and Hillchol OCVs had shown that it was well tolerated in mice while giving rise to good but not maximal antibody responses. An unvaccinated group of mice served as negative controls (Nil). Serum and fecal samples were collected 10–12 days after the last immunization.

All treatments and procedures for immunizations and sample collections were performed as described in previous studies [18] and were all in accordance with the Swedish Animal Welfare Act (1988:534) and the Animal Welfare Ordinance (1988:539). Humane endpoints were used to minimize animal suffering. Mice were monitored daily for the first three days after immunization for visual signs of adverse reactions (reduced activity or responsiveness, affected fur coat condition, and/or body posture) and then at least three days a week during each experiment, and body weight was recorded just before and two days after each completed round of administration. Animals were sacrificed if they had a weight loss of more than 10% or showed signs of apathy or loss of fur. The studies were approved by the Ethical Committee for Laboratory Animals in Gothenburg, Sweden (Ethical permit numbers 1453/18 and 0038/23).

### 2.9. Analytical Methods and Reagents

#### 2.9.1. Reference Materials Used as Standards

O1 LPS standard. Purified V. cholerae O1 LPS antigen containing equal proportions of Inaba and Ogawa LPS was prepared from the Hikojima strain MS1568 for use as a standard [10]. Its LPS content (ca 60% of the dry weight) was determined by the Purpald method as described [50].

rCTB standard. Highly purified rCTB for use as a standard was prepared as described [37,40]. Its rCTB content was determined by the BCA Protein Assay Kits protein assay (Pierce, Waltham, MA, USA). Purity was assessed to be 97% by SDS-PAGE and densitometry. Analytical scale testing by fast liquid protein chromatography (FLPC) showed a single protein peak consistent with a >95% pentameric structure.

#### 2.9.2. Purity, Cell Morphology, and Serotype and Biotype Tests

These properties were examined as described [38].

#### 2.9.3. Genotyping Methods

Genomic DNA was isolated from seed-lot strains using the Qiagen gDNA Kit (Qiagen, Hilden, Germany) according to the manufacturer’s instructions and sent to Eurofins genomics for NGS (Next Generation sequencing) analyses (Ebersberg, Germany).

Specific genetic markers were assessed by PCR, including the presence or absence of the *wbeT* gene (associated with the Ogawa or Inaba serotype) and the deletion of the ctxAB genes encoding cholera toxin. Primers were synthesized by Eurofins Genomics (Ebersberg, Germany) and are listed in the Supplementary File, where the PCR Standard Operating Procedure (SOP) is also described in Section B.

#### 2.9.4. Determination of Formaldehyde

Formaldehyde (FA) was measured using the Purpald reagent as described in Quesenberry and Lee [39].

#### 2.9.5. Inhibition ELISA for Determination of *V. cholerae* O1 LPS Antigen in Bacterial Suspensions and Vaccine Preparations

The method described in Sharma et al. was used [38]. For maximal precision, all assays were performed testing samples in triplicate on each of two plates and repeating the assay on a subsequent day; the mean value of the triplicates from each plate was determined, and the final value was then calculated as the mean value from the four plates.

#### 2.9.6. GM1-ELISA for Determination of rCTB Antigen

This assay was used as described [37,42] with the same test design as described above for the LPS determinations.

#### 2.9.7. SDS-PAGE with Densitometry and Western Blot for Testing Quantity and Purity of rCTB

Samples were denatured by heating at 95 °C for 7 min in reducing sample buffer (NuPAGE LDS 4×; Novex^®^, Life Technologies, Carlsbad, CA, USA) with the addition of NuPAGE™ Sample Reducing Agent (500 mM dithiothreitol), Novex^®^, Life Technologies). Defined amounts—2, 4, and 6 μg of denatured rCTB standard and ca. 5 μg denatured test sample, all added in 10 μL, were separated by SDS-PAGE in a 4–12% Bis-Tris Mini Gel (NuPage gels Novex^®^, Life Technologies), followed by staining with Coomassie brilliant blue. Stained bands were analyzed using an EZ imager and Gel Doc software from Bio-Rad (Gel Doc EZ, Bio-Rad Laboratories AB, Solna, Sweden): purity of the rCTB test sample-stained monomer band was defined as the percentage of the total staining, and the amount was calculated in relation to the rCTB standard samples.

#### 2.9.8. Immunological Analyses of Antibodies in Serum and Fecal Samples of Orally Immunized Mice

Sera from mice collected before and after immunizations were analyzed for IgG and IgA antibodies to *V. cholerae* O1 Inaba and Ogawa LPS antigens, as well as to CTB, and fecal extracts for IgA antibodies to these antigens using ELISA and GM1-ELISA methods as described [37,43]. Sera were also analyzed for vibriocidal antibodies against Inaba and Ogawa bacteria, as described [51].

#### 2.9.9. Statistics

Statistical multigroup comparisons of antibody titers were performed using one-way ANOVA with Holm–Šídák’s post-test compensation for multiple comparisons in the Prism software system GraphPad 10.6.00 (GraphPad Software Inc., San Diego, CA, USA); 2-sided adjusted *p* values < 0.05 were interpreted to be statistically significant. Coefficient of variation percentages (CV%) between replicate samples or replicate tests were estimated in the same Prism GraphPad program.

## 3. Results

Figure 1 outlines the main steps in the preparation of the DuoChol capsule OCV, which are referred to below.

### 3.1. Characterization and Growth Properties of MS1955 Inaba and MS1987 Ogawa Vaccine Strains

#### 3.1.1. Genotype and Serotype Stability

**Figure 2:**
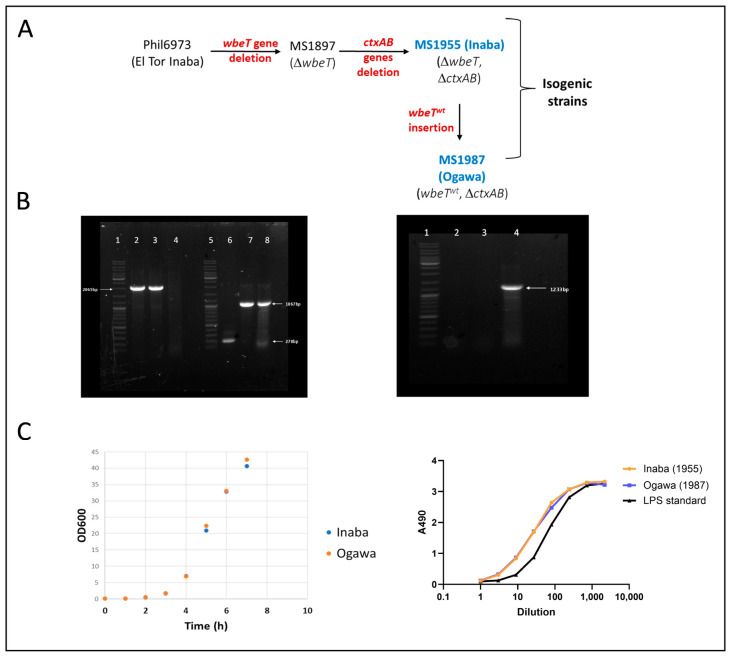
Construction and properties of isogenic El Tor *V. cholerae* O1 Inaba (MS1955) and Ogawa (MS1987) vaccine strains. (**A**) Construction of strains: The point mutated inactive *wbeT* gene in parental strain Phil6973 is removed, resulting in MS1897, followed by the deletion of the toxin-encoding genes ΔctxAB to give the MS1955 Inaba strain. The Ogawa serotype MS1987 strain is then generated by insertion of a wild-type Ogawa *wbeT* gene into the MS1955 strain. (**B**) PCR confirmation of CTX region and WbeT modifications in vaccine strains: **Left**: PCR using CTX primers (lanes 2,3,4) and WbeT primers (6,7,8). Lane 1: GeneRuler Ladder Mix; lane 2: Inaba MS1955 DNA; lane 3: Ogawa MS1987 DNA; lane 4: Phil6973 (Inaba) DNA. Lanes 2–3 show a ~2065 bp band indicating the expected deletion. No band is observed in lane 4, consistent with the larger amplicons > 10 kb not being amplified under the PCR conditions used. **Right**: PCR using CTXAB primers. Lane 1: GeneRuler Ladder Mix; lane 2: Inaba MS1955; lane 3: Ogawa MS1987; lane 4: Phil6973 (Inaba). No amplification is detected in lanes 2–3, confirming the absence of ctxA. Lane 4 shows a ~1233 bp band corresponding to the ctxAB genes. Lane 5, GeneRuler Ladder Mix¸ lane 6, Inaba MS1955; lane 7: Ogawa MS1987; lane 8: Phil6973 (Inaba). Lane 6 shows a small band of 278, indicating the expected deletion, and Lane 7 shows the WbeT gene amplification. Lane 8 shows that the WbeT gene product, although Phil 6973 is Inaba, its phenotype is due to a point mutation introducing a stop codon, so with this PCR, the product is indistinguishable from an Ogawa strain such as MS1987. (**C**) **Left**. Growth curves (OD_600nm_) for the Inaba (MS1955) and Ogawa (MS1987) strains during fermentation in rich medium for 7 h, showing indistinguishable bacterial growth. **Right**. O1 LPS antigen Inhibition-ELISA curves for formalin-inactivated Inaba and Ogawa strains showing their identical levels of O1 LPS antigen. The curves show the inhibitory activities of Inaba MS1955 and Ogawa MS1987 at different dilutions starting from bacterial density OD = 10 and of a purified O1 LPS standard at a starting concentration of 0.4 mg/mL; the O1 LPS antigen levels in the bacterial suspensions are determined by comparing their 50% inhibitory dilution with that of the LPS standard.

The generation of the MS1955 and MS1987 strains is summarized in Figure 2A. The strains are isogenic, differing only in the *wbeT* gene determining the serotype: MS1955 Inaba lacks *wbeT*, which is present in the MS1987 Ogawa strain. The parent Phil6973 strain has the Inaba serotype due to a point mutation in the *wbeT* gene. This mutated wbeT gene was deleted, resulting in the fully stable Inaba strain MS1897, from which the cholera toxin-encoding ctxAB genes were then further deleted to generate the MS1955 Inaba strain (∆wbeT, ∆ctxAB). The MS1987 Ogawa strain (wbeT^wt^, ∆ctxAB) was generated by insertion of a wild-type wbeT gene into the MS1955 strain.

PCR analyses verified the critical genetic modifications of the MS1955 (∆*wbeT*, ∆*ctxAB*) and MS1987 (*wbeT*^wt^, ∆*ctxAB*) strains (Figure 2B). The PCR analyses were repeated at intervals over more than 5 years and more than 50 subcultures, with results supporting genotypic stability. Bacterial slide agglutination with serotype-specific diagnostic sera also verified serotype stability over these many subcultures and years.

#### 3.1.2. Growth Properties

The isogenic MS1955 and MS1987 strains had indistinguishable growth properties. Thus, irrespective of the many growth media tested, the growth curves were identical both in shake flasks and in fermenters (Figure 2C, left panel). Indeed, even when the strains were mixed in different proportions and co-cultured, the relative proportions of the strains remained unchanged (Supplementary File, Section C). The amounts of O1 LPS antigen per OD unit of growth were also indistinguishable (Figure 2C, right panel).

### 3.2. Production and Purification of rCTB

#### 3.2.1. Characterization of the MMS1692 Production Strain

**Figure 3:**
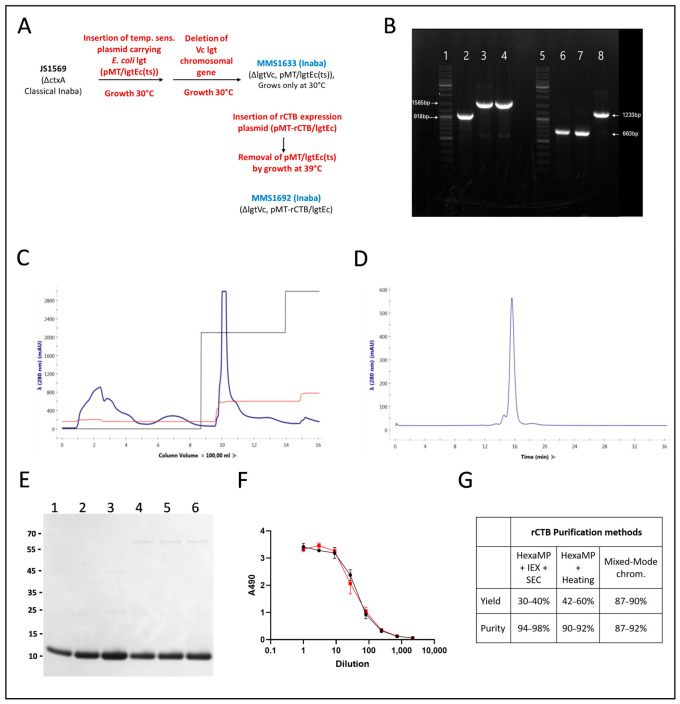
Development of rCTB-producing MMS1692 strain and of rCTB purification by mixed-mode chromatography. (**A**) Schematic illustration of the construction of MMS1692 from parental strain JS1569, a *ctxA* gene deleted derivative of classical biotype *V. cholerae* O1 Inaba wild type strain 569B. (**B**) PCR confirmation of lgtVc and ctx deletions in MMS1692. Lanes 1–4: PCR using lgtVc primers. Lane 1: GeneRuler Ladder Mix; lane 2: Inaba MMS1692; lane 3: JS1569; lane 4: 569B. Lane 2 shows a ~918 bp band consistent with deletion of the lgtVc region, whereas lanes 3 and 4 display a ~1585 bp band corresponding to the intact gene. Lanes 5–8: PCR using ctxAB primers. Lane 5: GeneRuler Ladder Mix; lane 6: Inaba MMS1692; lane 7: JS1569; lane 8: 569B. Lanes 6 and 7 show a ~663 bp band consistent with deletion of ctxA, while lane 8 displays a ~1233 bp band representing the full ctxAB operon. (**C**) Chromatogram of MMC purification of rCTB from MMS1692 culture medium. The material was loaded in binding buffer (BB) onto the column; the bound rCTB was then eluted using a step gradient with a 70% mixture of Elution Buffer (EB) and 30% BB and collected; a final 100% EB step completed the gradient. Blue: A280 nm protein; Black: Step gradient; Red: Conductivity. (**D**) Size-exclusion chromatography of MMC purified rCTB on a Superdex 200 26/60 column (Cytiva, Sweden) showing >95% of the protein migrating as pentameric rCTB protein. (**E**) Coomassie-stained SDS-PAGE gel after test under denaturing/boiling conditions showing ~95% purity of MMC purified rCTB. Lanes 1–3: highly purified rCTB standard in three concentrations; lanes 4–6: rCTB purified by MMC tested in triplicate. Total stained protein and purity were evaluated by densitometry using a Bio-Rad gel imager. Positions of molecular weight marker proteins run under similar conditions are indicated. (**F**) GM1 ELISA curves showing “identical” GM1 receptor-binding and antigenic properties of MMC-purified (red) and reference rCTB (black); starting protein concentration was 2 μg/mL (**G**) Recovery and purity comparisons of rCTB purified by alternative methods (each in 3–5 separate experiments): (i) “traditional” three-step method (Hexametaphosphate [HexaMP] precipitation + ion-exchange chromatography [IEX] + size-exclusion chromatography [SEC]); (ii) two-step method (HMPP followed by heat treatment at 65 °C for 30 min); and (iii) MMC single-step method. Recoveries were determined by analyzing rCTB concentrations before purification and after the final purification step using SDS-PAGE and densitometry, and the same method was also used to assess the purity of the final rCTB preparations.

rCTB was produced from the *V. cholerae* classical biotype O1 (Inaba) MMS1692 strain, which was engineered to express high levels of rCTB constitutively from a ctxB gene encoded under the control of the synthetic tac promoter on a high-copy-number plasmid. The construction of this strain is schematically summarized in Figure 3A. The plasmid is maintained by complementation of an essential gene, lgt, with the corresponding gene from *E. coli,* thus avoiding any need for use of an antibiotic to maintain the plasmid. The key genetic modifications of MMS1692, including the deletion of the chromosomal ctxA and lgt genes, were verified by PCR analyses (Figure 3B). Similar to MS1955 and MS1987, the genotypic and phenotypic properties of MMS1692 tested by PCR and serotyping methods have proved to be stable over more than 50 subcultures over more than 5 years.

#### 3.2.2. High-Yield Production and Purification of rCTB

We have previously described that the MMS1692 strain could produce up to 1 g of secreted rCTB per liter of growth medium during fermentation for 18–24 h in modified Syncase medium [40]. We can now report that adding 1.5% yeast extract to the medium and prolonging the fermentation to 48 h increased production to ≥1.5 g secreted rCTB/Liter (Supplementary File, Section D). These latter conditions were therefore used.

To further increase the cost-effectiveness of rCTB production, a new, high-recovery mixed-mode chromatography (MMC) rCTB purification method was developed. The MMC column allowed binding of at least 2 g of concentrated crude rCTB per 100 mL column resin. As illustrated in Figure 3C, the bound rCTB could then be completely recovered by step gradient elution. When the purified rCTB was analyzed by size exclusion chromatography (SEC), it migrated almost exclusively as a single peak CTB pentamer, only preceded by a minor (<10%) protein peak (Figure 3D). SDS-PAGE analyses with densitometric assessment of Coomassie-blue-stained material confirmed the high purity of MMC-purified rCTB (Figure 3E). Only 4–5% of the total stained protein was found beyond the rCTB monomer band. When the two main “contaminant” protein bands, at positions ~66 kDa and 24 kDa, from an overloaded SDS-PAGE gel were sent for mass spectrometric analysis, they were found to mainly be glutamine synthetase and CTB (probably linked subunit dimers), respectively.

When the purified rCTB protein was tested by GM1-ELISA, it proved to have identical activity as the reference rCTB, demonstrating intact GM1 receptor binding as well as rCTB oligomer-specific monoclonal antibody binding properties (Figure 3F). This was seen also for SEC column fractions analyzed by GM1-ELISA, when not only the rCTB pentamer protein from the predominant SEC peak but also fractions from the front of the faster migrating protein peak as well as a fraction preceding this peak were positive revealing the presence of larger rCTB oligomers with retained functionality in these fractions; indeed a total of more than 2.5% of the total GM1-ELISA activity was found in these fractions containing 4.7% of the total protein, suggesting that ca half of this faster migrating material was functionally active rCTB.

To compare the recovery of rCTB purified by MMC and previous methods, we undertook 3–5 separate studies of the recovery and purity of rCTB from MMS1692 culture filtrate using different purification methods: (1) Hexametaphosphate precipitation (HMPP) + ion exchange chromatography + SEC (N = 3); (2) HMPP + heat treatment at 65 °C (N = 3); and (3) MMC (N = 5). The results demonstrate a superior recovery, 87–95%, achieved with the MMC method compared to the other methods (Figure 3G).

### 3.3. Vaccine Lyophilization, Powder Preparation, and Capsule Filling

Previous work indicated that freeze-drying of formalin-killed *V. cholerae* O1 and rCTB mixtures with protective sugars like sucrose or trehalose can provide dry vaccines with retained antigens and good stability [37,52].

These results were confirmed and extended when mixtures of DuoChol whole cell and rCTB components were lyophilized together with a broad range of different excipients. A concentration of 60 mg sucrose (or trehalose) per ml of vaccine mixture, higher in previous studies [17,25], was found to be optimal.

A new type of size 1 enteric capsules (Enprotect, Lonza) was chosen for manual filling with the DuoChol vaccine powder prepared after lyophilization. These capsules have been described by the manufacturer and others [53] to be resistant at acid pH and to rapidly dissolve at pH ≥ 5.6, properties which we could confirm also using vaccine capsules filled with DuoChol OCV vaccine powder (Supplementary File, Section E).

**Figure 4:**
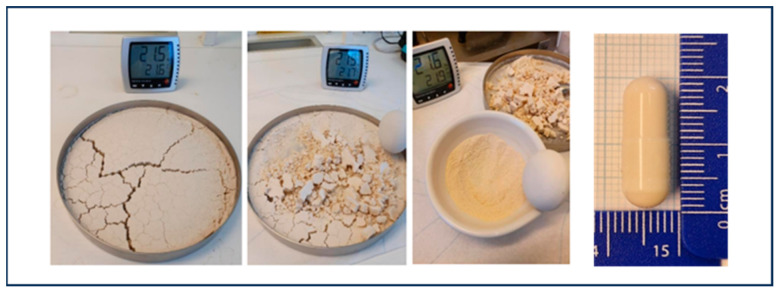
Freeze-dried “vaccine cake” which, by crushing using pestle and mortar, generates the vaccine powder which, after sieving, is filled in size 1 enteric capsules as DuoChol DP OCV.

Figure 4 illustrates the steps for generating the vaccine powder used for filling the capsules: It shows a typical DuoChol OCV “vaccine cake” after lyophilization, which, after transfer to a mortar, is crushed with a pestle, generating the powder, which, after sieving, is filled in the size 1 Enprotect capsules. The cake was fragile, and gentle crushing was sufficient to generate a free-flowing powder, which, before being filled into capsules, was stored in an airtight bottle filled with N2 to prevent moisture uptake. When the vaccine powder was dissolved to the original liquid mixture concentration and compared side-by-side with the liquid mixture, there was no difference in either OD_600nm_ or in O1 LPS or CTB antigen contents between the dissolved powder and the liquid preparations (Table 1); as well, there was no difference in cell morphology as analyzed by light microscopy.

### 3.4. Properties of Duochol OCV Capsules and Stability During Storage at Different Temperatures

A DuoChol OCV lyophilized powder lot was used to fill DuoChol OCV prototype capsules. These were then stored at 4 °C, 25 °C, or 40 °C and analyzed within one week from preparation (time 0) and again after 2, 4, 6, and 21 months. Vials with dry powder from the same preparation that had not been filled into capsules and kept at 4 °C were used as reference material.

**Figure 5:**
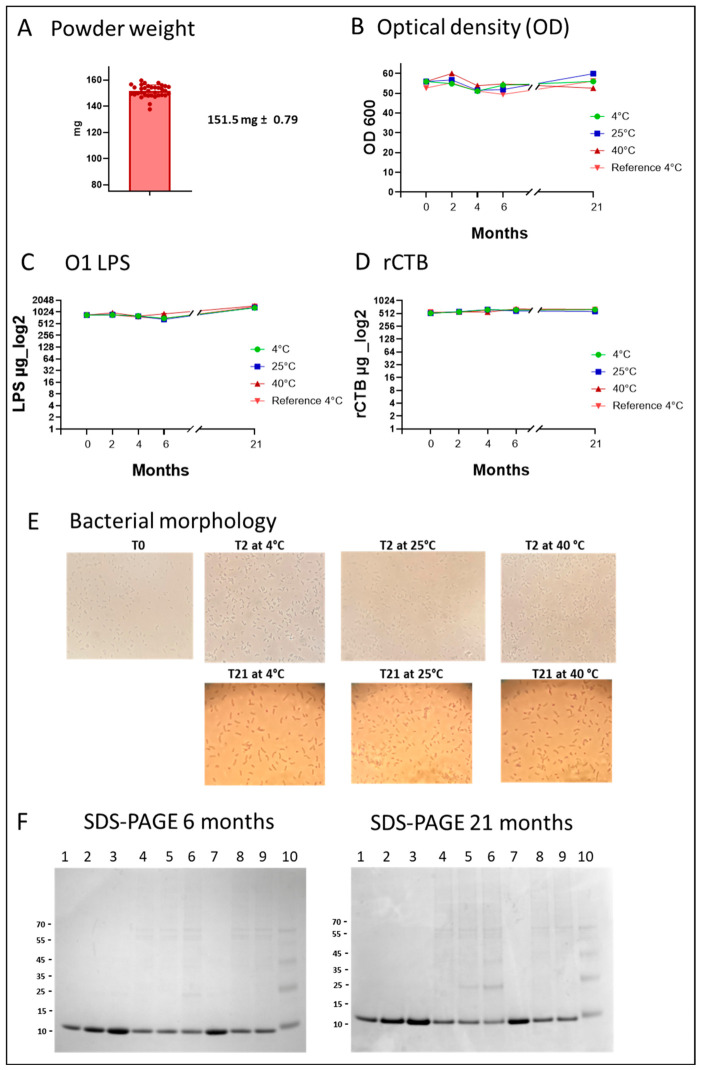
Stability of freeze-dried DuoChol OCV (WC + CTB vaccine in enteric capsules) stored under different conditions. (**A**) Powder weight of capsule contents was monitored during the stability program and remained stable over time, irrespective of storage temperature, 4–40 °C; 100 mg of pre-weighed samples of unencapsulated lyophilized powder stored side-by-side with the capsules throughout the study were used as a reference. Capsule contents and reference samples were resuspended in PBS (100 mg/mL) and analyzed for: (**B**) optical density at 600 nm (OD600); (**C**) LPS O1 antigen content by LPS inhibition ELISA; (**D**) rCTB content by GM1 ELISA; and (**E**) bacterial morphology by phase-contrast microscopy. (**F**) SDS-PAGE analysis of rCTB in DuoChol. After 6 and 21 months of storage at 4 °C, 25 °C, or 40 °C, samples were centrifuged at 8000 rpm for 10 min, and the supernatants were analyzed by SDS-PAGE under denaturing conditions (boiled at 95 °C for 5 min) followed by Coomassie staining; 5 µL of supernatant was loaded onto gels. Lanes 1–3: purified rCTB (2, 4, and 6 µg); lanes 4, 8, 9: capsules stored at 4 °C; lane 5: capsule stored at 25 °C; lane 6: capsule stored at 40 °C; lane 10: Dukoral stored at 4 °C (positive control). Distinct CTB oligomer bands were observed and increased with storage time in Dukoral (lane 10) but were not seen in DuoChol capsules stored at 4 °C for either 6 or 21 months (lanes 4, 8, 9) or in capsules stored at 25 °C for 6 months (lane 5). Minimal rCTB oligomer formation (limited to the dimeric band) was seen in capsules stored at 40 °C for 6 and 21 months, with no change in intensity between 6 and 21 months and in capsules stored at 25 °C for 21 months. No error bars are shown for the displayed mean values of three capsules each per assay (**B**–**D**) since they would be too small to be seen; indeed, the highest coefficients of variation (CV) noted were a mean CV of 6.5% (max 18.7%) for the LPS determinations (**C**) and 5.6% (maximum 11.3%) for the rCTB determinations (**D**).

The dissolved lyophilized powder contents of individual capsules were examined for bacterial density (OD_600nm_), O1 LPS and rCTB antigen contents, and microscopic appearance. The results are shown in Figure 5. Irrespective of storage time and temperature, the vaccine powder weight of the capsules, 151.5 ± 0.79 mg, varied only marginally (Figure 5A). Likewise, both OD_600nm_ (Figure 5B) and the O1 LPS (Figure 5C), and rCTB antigen contents (Figure 5D), remained stable at different temperatures during the entire 21-month study period.

Light microscopy showed insignificant cell clumping and an almost uniform, characteristic *V. cholerae* comma-shaped cell morphology over the whole period (Figure 5E). The vaccine powder stored at 25 °C and 40 °C had identical properties as the Reference powder stored at 4 °C at each of the tested time points.

We know that formalin, even in the low residual concentrations of all current inactivated OCVs, may cause crosslinking of B subunits within CTB pentamers. We noted in Dukoral OCV, after the supernatant had been isolated by removing cellular material by centrifugation, denatured by heating in reducing buffer, and examined by SDS-PAGE, in addition to the predominant rCTB monomer band, a few additional, progressively fainter bands with sizes consistent with dimers and trimers of CTB subunit monomers. These bands were more prominent in Dukoral lots with short time to expiry than in younger lots; yet, when the different lots were examined by GM1-ELISA, and compared with a purified rCTB standard, they had indistinguishable and, relative to the standard, full activity. These SDS-PAGE findings with Dukoral also made us examine the extent of B-subunit oligomer formation over time in DuoChol (Figure 5F). After 6 and 21 months of storage at 4 °C, 25 °C, or 40 °C, samples were centrifuged at 8000 rpm for 10 min, and the supernatants were analyzed by SDS-PAGE under denaturing conditions, followed by Coomassie staining. No CTB oligomerization was seen in DuoChol capsules stored at 4 °C for either 6 or 21 months or in capsules stored at 25 °C for 6 months. A very low level of oligomer formation (limited to the dimeric band) was seen in capsules stored at 40 °C for 6 and 21 months and in capsules stored at 25 °C for 21 months. Similar to Dukoral, the low level of oligomer formation in DuoChol (lower than that in Dukoral), clearly did not influence the GM1-ELISA activity, which, as seen in Figure 5D, was unchanged over the whole study period.

Although only one batch of capsules, and only three capsules for each storage temperature and time point, were analyzed in this prolonged stability study, results for individual data points showed very small variations between triplicate samples as specified in Figure 5 legend. A second batch of capsules is currently under stability testing and has to date proved to be stable for at least 6 months.

### 3.5. Systemic and Mucosal Immunogenicity of DuoChol OCV in Mice

The ability of the DuoChol OCV to induce an effective immune response against the key protective antigens LPS and CTB, also after long storage at different temperatures, was tested in orally immunized mice and compared with the immune responses to Dukoral OCV.

**Figure 6:**
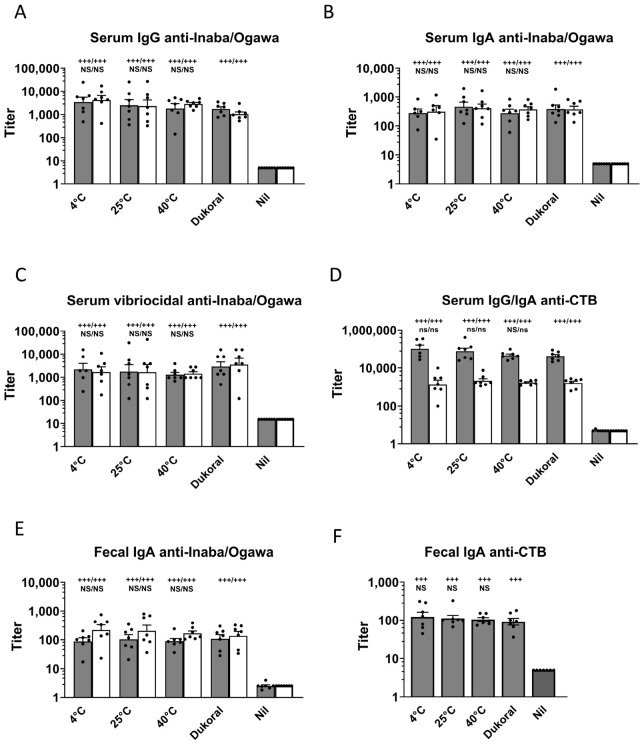
Serum and intestinal-mucosal antibody responses after oral immunizations with DuoChol vaccine stored at different temperatures for 5 months. Serum and fecal/mucosal antibacterial and antitoxic responses in mice were measured after oral/intragastric immunizations with dissolved DuoChol vaccine capsules stored at 4 °C, 25 °C, or 40 °C for 5 months or for comparisons with cold-chain stored Dukoral^®^ OCV. An unvaccinated group of mice served as negative controls (Nil). The mice (seven females per group) were immunized in three rounds at 2-week intervals, with 1/20th of a human dose given in each round. Serum and fecal samples were collected 10–12 days after the last immunization. Immune responses against LPS O1 (Ogawa and Inaba) and rCTB were measured by ELISA—serum was also tested for vibriocidal antibodies against Inaba and Ogawa bacteria—and are expressed as geometric means + SEM antibody titers: (**A**) Serum IgG anti-Inaba LPS (gray) and anti-Ogawa LPS (white); (**B**) Serum IgA anti-Inaba LPS (gray) and anti-Ogawa LPS (white); (**C**) Serum vibriocidal anti-Inaba (gray) and anti-Ogawa (white); (**D**) Serum IgG (gray) and IgA (white) anti-CTB; (**E**) Fecal IgA anti-Inaba LPS (gray) and anti-Ogawa LPS (white); and (**F**) Fecal IgA anti-CTB antibodies. Statistical differences for vaccinated groups versus the unvaccinated Nil group analyzed by one-way ANOVA with Holm-Šídák’s post-test compensation for multiple comparisons are shown in the upper row on top of the columns and are designated +++ for *p* < 0.001. No statistical differences were found between any of the vaccinated DuoChol groups and the Dukoral group, which is indicated as NS = Not significant (*p* > 0.50) or ns (*p* = 0.20–0.50) in the lower row on top of the columns.

**Figure 7:**
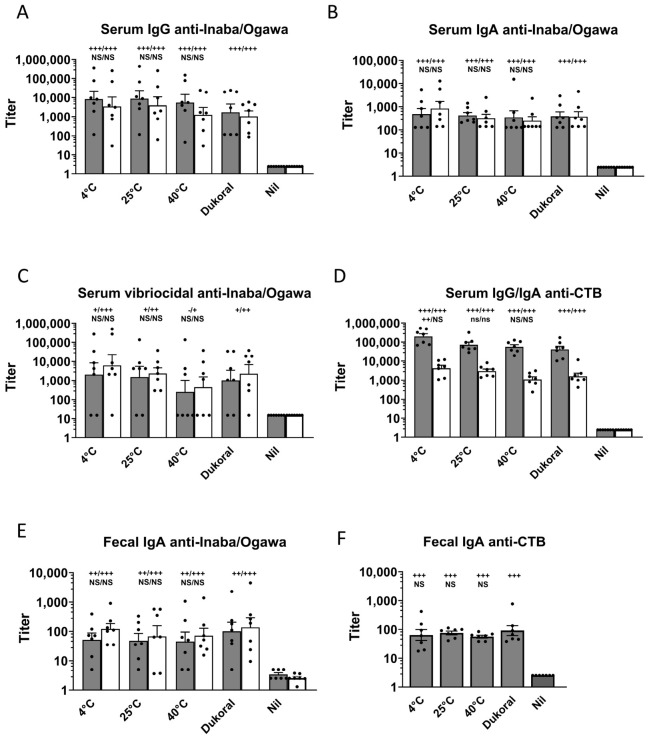
Serum and intestinal-mucosal antibody responses after oral immunizations with DuoChol vaccine stored at different temperatures for 21 months. For further information on the study design, see legend to Figure 6. Statistical differences for vaccinated groups versus the unvaccinated Nil group analyzed by one-way ANOVA with Holm-Šídák’s post-test compensation for multiple comparisons are shown in the upper row on top of the columns and are designated +++ for *p* < 0.001, ++ for *p* < 0.01, + for *p* < 0.05, and – for *p* = 0.11. No statistical differences were found between any of the vaccinated DuoChol groups and the Dukoral group, which is indicated as NS = Not significant (*p* > 0.50) or ns (*p* = 0.20–0.50) in the lower row on top of the columns.

The serum antibody results are presented in Figure 6A–D (5 months storage) and Figure 7A–D (21 months storage). All of the vaccinated groups displayed strong responses in all types of antibodies compared to unvaccinated controls (all with *p* values <0.001 except for vibriocidal antibody responses after 21 months of storage, where significances versus the Nil group were lower and for one group absent). No significant differences were seen between any of the vaccinated groups, including the group immunized with Dukoral (all with *p* > 0.20 and most with *p* > 0.50).

Likewise, as illustrated in Figure 6E,F and in Figure 7E,F, fecal IgA antibody responses to all of Inaba LPS, Ogawa LPS, and CTB antigens were strongly elevated in all vaccine groups compared to the unvaccinated controls. *p* values were <0.001 for all groups and types of antibodies at 5 months and <0.001 or <0.01 for responses after 21 months. Further, similar to the serum antibody responses, no significant differences were seen between any of the vaccines, irrespective of their storage temperatures or times, including Dukoral stored at 4 °C, in their ability to induce intestinal-mucosal fecal IgA antibodies to either Inaba or Ogawa LPS or CTB (*p* > 0.50 for all these group comparisons).

## 4. Discussion

Oral cholera vaccines (OCVs) have been described by the WHO Global Task Force for Cholera Control (GTFCC) as a “game changer” for cholera prevention, yet global supply continues to fall short of demand. A major constraint of current OCVs is their dependence on cold-chain storage, which complicates logistics, increases delivery costs, and contributes to vaccine wastage. Thermostable OCVs—particularly those stable at temperatures up to 40 °C—could substantially simplify distribution and expand access in the low-resource settings where cholera burden is greatest [54].

In this context, we developed DuoChol, a thermostable, capsule-based OCV containing lyophilized, formalin-inactivated *V. cholerae* O1 Inaba and Ogawa strains together with recombinant cholera toxin B subunit (rCTB). Inclusion of rCTB is known to enhance short-term protection against cholera and, as well, to confer cross-protection to diarrheal disease caused by enterotoxigenic *Escherichia coli* (ETEC) producing LT-like toxins [36,55]. The inclusion of rCTB in an enteric capsule in the DuoChol formulation thus offers potential for improved efficacy compared to inactivated whole-cell only OCVs. Compared to the Dukoral OCV, which also contains rCTB, the DuoChol formulation may offer comparable immunogenicity without any need for buffer co-administration, a practical benefit especially in mass vaccination campaigns.

A central achievement of this work is the generation of the isogenic whole-cell vaccine strains MS1955 (Inaba) and MS1987 (Ogawa). These strains differ solely in the presence or absence of a functional *wbeT* gene and have exhibited excellent long-term genetic and phenotypic stability. Their indistinguishable growth characteristics—including the ability to be co-cultured to high density without shifts in relative abundance—indicate an absence of competitive fitness differences, an advantage for large-scale vaccine manufacturing where consistent antigen output is important.

The rCTB-producing strain MMS1692 similarly demonstrated high genetic stability and robust expression of secreted rCTB. Its design, based on complementation of the essential *lgt* gene to maintain a high-copy plasmid, enabled modifications of the growth and feeding media not possible with classical auxotrophic systems. The addition of yeast extract, combined with extended 48 h fermentation, increased yields to approximately 1.5 g/L of secreted pentameric CTB. Further, development of a new, scalable mixed-mode chromatography (MMC) purification process resulted in nearly 90% recovery of rCTB with high purity, correct oligomerization, and full GM1-binding activity. These advances have important implications for affordability and industrial scalability.

The dry, capsule-based formulation represents another major innovation. Systematic evaluation of excipients and drying methods demonstrated that lyophilization with adequate concentrations of sucrose or trehalose preserves antigen integrity, minimizes residual moisture, prevents bacterial clumping, and produces a powder with excellent flow properties for capsule filling. The selected enteric capsules reliably resist gastric acidity and dissolve rapidly above pH 5.6, ensuring targeted small-intestinal release, as also supported by recent clinical tests with other materials [53,56]. Antigen content, cell morphology, and physical characteristics remained unchanged upon reconstitution.

DuoChol’s thermostability profile is particularly important. Capsules stored for up to 21 months at 4 °C, 25 °C, or 40 °C retained O1 LPS and rCTB antigen content, optical density, and bacterial morphology. Only minimal aggregation was observed after prolonged storage at 40 °C, without loss of antigenicity. Such stability at elevated temperatures has not been reported for any of the currently available OCVs and represents a major advantage for deployment in cholera-endemic regions with limited cold-chain capacity. Thermostability would enable local stockpiling and more rapid outbreak response.

The functional stability of DuoChol was further confirmed in vivo. In mice, oral immunization elicited strong vibriocidal responses and robust serum IgG and IgA anti-LPS and anti-CTB antibodies, similar to those induced by the licensed OCV Dukoral. Likewise, fecal IgA antibody responses to both O1 LPS and rCTB were also indistinguishable from those obtained with Dukoral, supporting the mucosal immunogenicity of the capsule formulation. Complementary studies in pigs have confirmed capsule integrity, intestinal release, and both systemic and mucosal immunogenicity after swallowing of whole DP capsules twice or three times at 2-week intervals (Devriendt B, Cox E, Terrinoni M, Lebens M, Holmgren J, to be published). There is no useful small animal model for assessing the protective immunogenicity of OCVs, since available infection challenge models are limited to baby mice or rabbits too young to mount an immune response. Therefore, the evaluation of non-inferior immunogenicity of a new vaccine candidate compared side-by-side with the closest similar licensed OCV—in our case Dukoral, which contains both inactivated Inaba and Ogawa whole cells and rCTB—is to date the best predictive evaluation of likely future protective immunogenicity in humans, especially when, as done in this study, not only serum antibody responses but also intestinal-mucosal IgA responses are compared. Indeed, also in humans, the currently available OCVs licensed from Euvichol and after (Euvichol-Plus, Euvichol-S, and Hillchol) were regulatory approved based on their non-inferior serum vibriocidal antibody responses to side-by-side tested Shanchol OCV without previous efficacy testing.

Engagement with public-health stakeholders in Bangladesh, Ethiopia, Kenya, Mozambique, and Tanzania revealed strong enthusiasm for an OCV with DuoChol’s characteristics. Thermostability and capsule format were identified as the most valuable features, addressing long-standing barriers to vaccine delivery [57]. Self-administration—particularly for booster doses—was viewed as feasible with appropriate instructions, suggesting potential for more flexible, community-driven delivery strategies [57].

Despite these advances, several challenges remain: One is that the described vaccine production was limited to laboratory scale. Another could be that thermostabilization steps, such as lyophilization and capsule formulation, may increase manufacturing costs. However, modeling studies indicate that savings from simplified logistics and reduced cold-chain requirements typically offset these expenses, making thermostable vaccines generally cost-effective even at two- to three-fold increased production costs [58]. Our projections—although admittedly preliminary and based on our previous experience with up-scaling to industry use of licensed inactivated OCVs Dukoral, OrcVax/Shanchol, and Hillchol—suggest that large-scale DuoChol manufacturing could achieve cost parity with existing liquid OCVs. A third challenge is the common inability of capsule swallowing in children under 4–5 years of age. Optimal solutions to this problem may vary by context and will require further evaluation.

## 5. Conclusions

In conclusion, our findings demonstrate that DuoChol is a promising, stable, and scalable OCV candidate with the potential to reshape cholera control strategies by addressing both supply constraints and cold-chain limitations. GMP manufacturing is underway, and phase 1 clinical testing is planned for 2026.

## Figures and Tables

**Table 1:** Comparison of bacterial optical density and O1 LPS and rCTB antigen contents in liquid and resuspended lyophilized powder preparation (mean ± SEM, triplicate testing).

	OD_600nm_	O1 LPS µg/mL	rCTB µg/mL
Liquid	62.5 ± 0.5	1037 ± 16.3	1550 ± 7.2
Dry powder	60.1 ± 1.1	1224 ± 0.67	1553 ± 75.2

## Data Availability

The original contributions presented in this study are included in the article/Supplementary Materials. Further inquiries can be directed to the corresponding authors.

## Supplementary Materials

The following supporting information can be downloaded at: https://www.mdpi.com/article/10.3390/vaccines14070573/s1.
