# Supplementary material for: Development of DuoChol, a Thermostable Inactivated Whole-Cell/B-Subunit Oral Cholera Vaccine in Enteric Capsule
Source: Vaccines (Basel). 2026 Jun 29;14(7):573. doi: 10.3390/vaccines14070573 (PMC13416707; doi:10.3390/vaccines14070573)

Duochol Supplemental file

Table of Contents

Section A. Construction, origins and properties of Duochol vaccine strains MS1955 (Inaba), MS1987 (Ogawa) and MMS1692 (rCTB) 2

Section B. PCR assay for confirmation/identification of *V. cholerae* strains MS1955, MS1987 and MS1692 29

Section C. Potential for co-culture of isogenic Inaba and Ogawa vaccine strains ..37

Section D. Confirmation of stability at acid pH and rapid dissolution at neutral pH of Enprotect enteric capsule used for DuoChol OCV 40

Original images from the main Manuscript...43

Section A. Construction, origins and properties of Duochol vaccine strains MS1955 (Inaba), MS1987 (Ogawa) and MMS1692 (rCTB)

1. Vaccine strains MS1955 and MS1987

1.1. Introduction.

MS1955 and MS1987 are isogenic derivatives of the El Tor O1 *Vibrio cholerae* strain Phil6973. The latter strain is a formalin-killed component of all WHO prequalified oral cholera vaccines (OCV) -- Dukoral™, Shanchol™ and Euvichol-Plus™/Euvichol-S™-- and is also the parent strain for the Hillchol™ formalin-inactivated whole-cell OCV recently licensed in India .

The Phil 6973 strain is an early 7th pandemic clinical isolate from the Philippines, which was received at the Univ of Gothenburg (UGOT/Dr Jan Holmgren et al.) from the Statens Serum Institute in Copenhagen in 1970/71. After confirmation of its purity and identity (El Tor biotype and Inaba serotype) the Phil6973 strain has been kept frozen at -70^{0}C in glycerol. A copy of the strain was transferred from UGOT to the Statens Bacteriological Laboratory in Stockholm (SBL) in 1983/84 and has been used from then in the development and manufacturing of the Dukoral OCV. A copy of Phil6973 (and of the two classical biotype strains Cairo 48 [Inaba]and Cairo 50 [Ogawa] in Dukoral as well as a V. cholerae O139 strain, 4260B) was later also provided from UGOT to NIHE in Vietnam where Phil6973 and these other strains were used in the production of Vietnam´s OrcVax/ mOrcVax OCVs and later also in the manufacturing of the Shanchol (India) and Euvichol (S. Korea) OCVs.

The Duochol vaccine strains MS1955 and MS1987 were generated so that their serotypic phenotypes will be stable. Phil6973 has the Inaba serotype due to a point mutation in the *wbeT* gene. The gene product of this gene is a methyl transferase that methylates the terminal sugar of the surface lipopolysaccharide (LPS) O1-antigen. Thus, strains of O1 *V. cholerae* with an intact *wbeT* gene have a methylated surface LPS giving rise to the Ogawa serotype. When the gene is inactivated the LPS is no longer methylated, and the resulting strains have the Inaba serotype. Point mutations such as that in the Phil6973 *wbeT* may revert to wild type by a point mutation. To remove

this possibility the *wbeT* gene was deleted from the chromosome entirely (MS1955, Inaba) and then a "wildtype" fully intact wbeT gene was introduced into this strain to generate the MS1987 Ogawa strain. Thus, the two whole cell bacteria strains are isogenic except for the *wbeT* gene responsible for adding a methyl group on the terminal perosamine of the surface lipopolysaccharide, that determines the serotype, which is deleted in Inaba and present in Ogawa. Both bacterial strains are also ctxAB deleted, which is different from the parent strain, and they therefore cannot produce cholera toxin as an extra safe-guard for their safety for use in a vaccine. Furthermore, the removal of the cholera toxin-encoding genes makes the strains less hazardous to handle in the vaccine production processes. A summary overview of the construction of MS1955 and MS1987 is illustrated in Figure S1

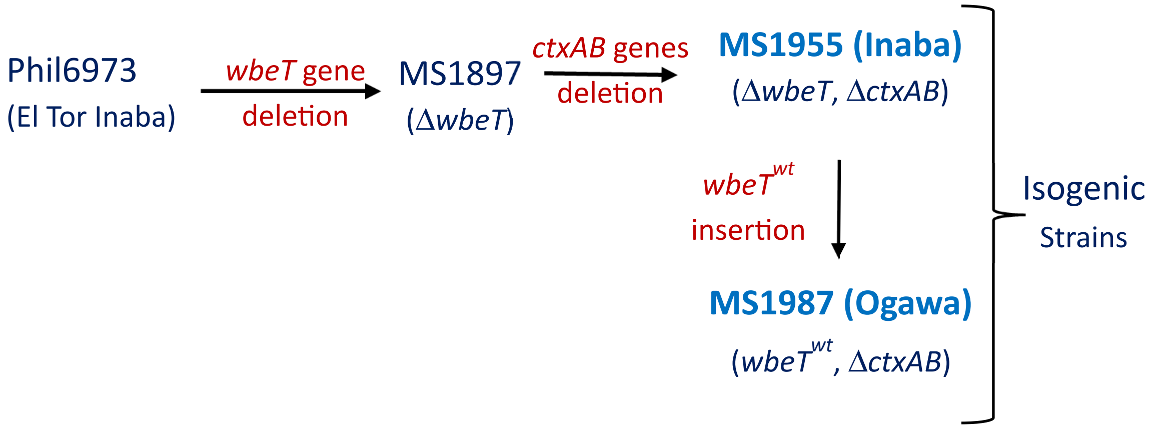

Figure S1. Summary overview of genetic construction of MS1955 and MS1987

1.2. Construction of MS1955 (Inaba)

- **Deletion of the wbeT gene from Phil6973.**

1. Plasmid construction: Point mutations that inactivate genes either by introducing a stop codon or by causing an amino acid change can revert and, although under the growth conditions used this is a rare event; to remove this possibility altogether, the *wbeT* gene was deleted from the chromosome completely. This was achieved using the R6K-based suicide vector pML-ssB generated in this laboratory. The pML-ssB plasmid carries a chloramphenicol resistance marker, an origin of transfer (*oriT*) and the *Bacillus subtilis sacB* gene conferring sucrose sensitivity to gram negative bacteria. It can only replicate in bacteria that can provide the R6K *pir* gene *in trans*. In other backgrounds it cannot replicate.

Flanking sequences on either side of the *wbeT* gene (500bp) were amplified from chromosomal template DNA obtained from Phil6973. The primers inserted a linker region containing SalI and EcoRV sites at the ends adjacent to the region to be deleted and XbaI and XhoI sites at the other ends.

The homology of the linker region on the two amplified fragments meant that they could be joined by primerless PCR. The resulting 1kb fragment could then be cloned into the pML-ssB vector by digestion with XbaI/XhoI and ligation into plasmid DNA digested with the same enzymes. Ligated DNA was transformed into *E. coli* strain MFDpir which carries the R6K *pir* gene required for plasmid replication as well as the genes allowing transfer of the plasmid to other strains by conjugation. The strain is also *dapA*$^{-}$ and therefore requires supplementation of the growth medium with diaminopimelic acid.

Transformants were selected by acquisition of chloramphenicol resistance on LB agar plates supplemented with 0.3mM diaminopimelic acid and chloramphenicol. Clones carrying the insert were identified first by PCR and subsequently by DNA restriction analysis of purified plasmid and DNA sequencing. The resulting plasmid was pML-ssB/wbeTΔ.

To provide a marker for counter-selection in *Vibrio cholerae,* a SalI/EcoRV cassette carrying the Tn5 kanamycin resistance gene flanked by *loxP* sites for Cre-mediated recombination was inserted into SalI/EcoRV-digested pML-ssB/wbeTΔ. Ligated DNA was transformed into *E. Coli* MDFpir and transformants were selected on LB agar plates supplemented with kanamycin and diaminopimelic acid. Correct plasmids were detected by PCR followed by DNA restriction analysis of purified plasmid. The final plasmid was pML-ssb/wbeTΔ(Km^{R}).

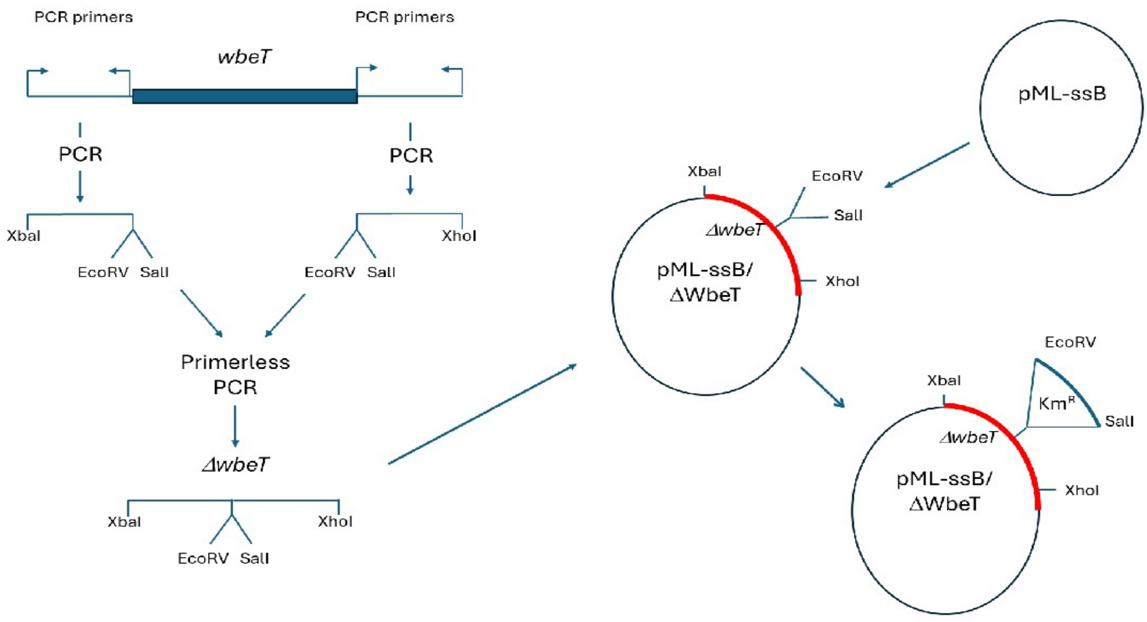

Figure S2. Construction of suicide vector for removal of the mutant wbeT gene from Phil 6973.

2. Conjugation and deletion of the wbeT gene from Phil6973: The pML-ssb/wbeTΔ(Km^R) plasmid was used to delete the *wbeT* gene from Phil6973 by a gene replacement procedure. The plasmid was introduced into Phil6973 by conjugation from the *E. coli* MFDpir host. The conjugation was performed on an LB-agar plate supplemented with diaminopimelic acid only. Cells of both donor and recipient strains were patched onto the same plate and allowed to grow overnight at 37° C.

Cells were then taken from the mating plate and streaked out on LB agar plates supplemented with kanamycin only. Under these conditions only the recipient Phil6973 cells will be able to grow if they have acquired the suicide vector together with the *V. cholerae* DNA carrying the *wbeT* deletion. Since the vector cannot replicate in the absence of the *pir* gene, the only way recipients could acquire the km^R phenotype was by insertion of the entire plasmid into the chromosome by homologous recombination. Transconjugants were therefore resistant to both kanamycin and chloramphenicol. Furthermore, the cells carried two copies of the *wbeT* region, one carrying the *wbeT* gene and the other carrying the kanamycin resistance marker. The two copies are separated by the plasmid DNA.

Spontaneous recombination occurs between the duplicated regions of the chromosome resulting in the excision of the plasmid and the loss of chloramphenicol resistance. This can result in retention of the *wbeT* gene or its replacement with the

kanamycin cassette. In order to select for the latter possibility, cultures were maintained on medium supplemented with kanamycin.

To select for strains that had lost the plasmid through homologous recombination, cultures were passaged in liquid LB medium supplemented with kanamycin with shaking at 37° C for two days. Aliquots of the resulting cultures were then plated out on modified LB agar plates lacking salt and supplemented with kanamycin and 12% sucrose. The plates were incubated overnight at 37° C. Under these conditions the *B. subtilis sacB* gene is lethal and acts as a negative selection against cells that have retained the plasmid in the chromosome. Colonies that grew were checked for sensitivity to chloramphenicol and screened for the correct genotype by PCR.

3. ***Removal of the Kanamycin resistance gene.*** Having removed the wbeT gene from the chromosome, the kanamycin gene that was inserted in its place was also removed by Cre mediated recombination. As described, the kanamycin resistance is flanked by tandem loxP sites. The strain was therefore transformed with a commercially obtained temperature-sensitive plasmid in which the *cre* gene is expressed from the *araBAD* promoter under the control of the araC repressor (p705-cre). The cells were transformed with the plasmid which confers chloramphenicol resistance and selected and maintained at 30° C. In order to remove the kanamycin resistance gene, the resulting strain was grown up overnight in liquid LB medium at 39° C in the presence of 0.2% arabinose and in the absence of any antibiotic supplements. The cell suspension was then plated out to single colonies on LB agar plates.

Colonies were subsequently screened for sensitivity to both chloramphenicol and kanamycin. Colonies with the correct phenotype were then screened for the chromosomal deletion of the *wbeT* gene by PCR and DNA sequencing. The final *wbeT* deleted strain was MS1897.

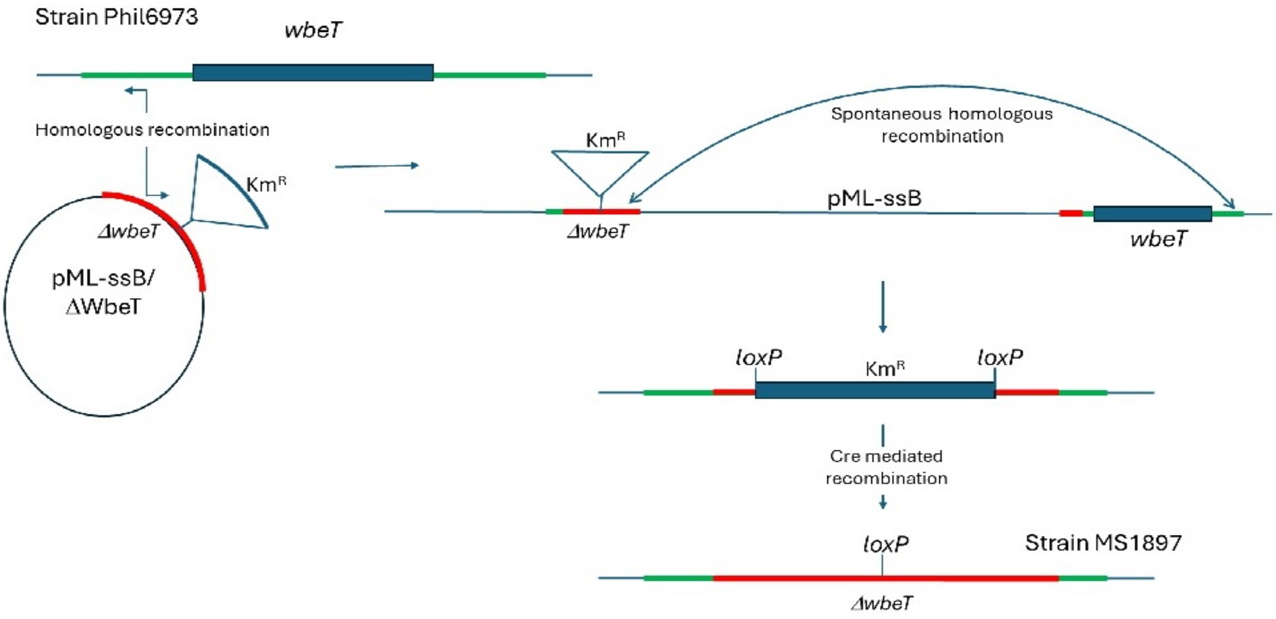

Figure S3. Stages in the removal of *wbeT* from the chromosome of Phil6973; The Suicide vector integrates into the chromosome by homologous recombination. Spontaneous homologous recombination removes the wbeT gene leaving the KmR gene. The KmR gene is removed by Cre-mediated recombination.

- **Removal of *ctxAB* cholera toxin genes from the *ΔwbeT* strain MS1897.**

The purpose of removing the cholera toxin gene from MS1897 was to generate an O1 *V. cholerae* vaccine strain that was safer to handle than the original virulent clinical isolate Phil6973.

The approach was to use a recombineering strategy in which loxP-Cre driven recombination deletes the relevant region of the chromosome. Two suicide vectors were made in order to introduce loxP sites into the chromosome on either side of the region containing the CTXΦ phage sequence(s) (figure S3). The region between the two sites was then removed by Cre-mediated recombination. In this case the *cre* gene was introduced on a plasmid carrying ampicillin resistance rather than chloramphenicol resistance since the strain from which the CTXΦ was deleted still contained a copy of the suicide vector in the chromosome and was therefore already chloramphenicol resistant.

1. Construction of suicide vectors for introduction of loxP sites on either side of the CTXΦ region. The removal of the CTXΦ region from chromosome I of V. cholerae MS1897 involved the construction of two suicide vectors designed to introduce loxP sites on either side of the region. Since there could be several tandem repeats of CTXΦ the amplified regions on either side of the locus were chromosomal fragments with no homology with the region to be deleted.

pML-ssB-ΔCTX1. The rationale behind the construction of this plasmid is shown in figure 3. Firstly, a Km^R gene was inserted into the suicide vector pML-ssB. The strains used to perform the cloning were those already described earlier. Subsequently a 1kb fragment was amplified from immediately downstream of the CTXΦ region of MS1987 with primers that introduced *loxP* sites on either side of the insert. This fragment was inserted into the pML-ssN(Km^R) plasmid adjacent to the Km^R gene. The plasmid was then inserted into the chromosome of MS1987 by patch mating as described earlier, selecting transconjugants for acquisition of kanamycin resistance. A plasmid carrying the *cre* gene was then introduced into the resulting strain and used to delete the plasmid and the Km^R gene by Cre-mediated recombination. This resulted in a loss of kanamycin and chloramphenicol resistance and introduced a single l*oxP* site flanked by a 1kb repeat of the cloned chromosomal region (strain MS1897a). See figure S3A.

pML-ssB-ΔCTX2. A second suicide plasmid was constructed in which a 1Kb fragment was amplified from the chromosome upstream of the CTXΦ region. In this case the amplified fragment was engineered so that it could be cloned adjacent to the Km^R gene in pML-ssb(Km^R) with the amplified fragment and the Km^R gene flanking a single *loxP* site (see figure S3B). This plasmid was transferred into strain MS1897a and integrated into the chromosome by homologous recombination and conferred kanamycin and chloramphenicol resistance (strain MS1897b).

2. Cre-mediated recombination to remove the CXTF region. Finally, a plasmid carrying the *cre* gene was transformed into MS1897b. Expression of the *cre* gene drove recombination between the two *loxP* sites resulting in the loss of kanamycin and chloramphenicol resistance and the entire CTXΦ region.

Strains that were sensitive to kanamycin and chloramphenicol were subjected to PCR analysis and DNA sequencing to confirm the absence of *ctxAB* genes. The sequencing confirmed not only that the *ctxAB* genes were absent, but also that the single *loxP* site resulting from the Cre-mediated recombination was present (see figure S3B).

The *ctxAB*-deleted strain is **MS1955**.

A

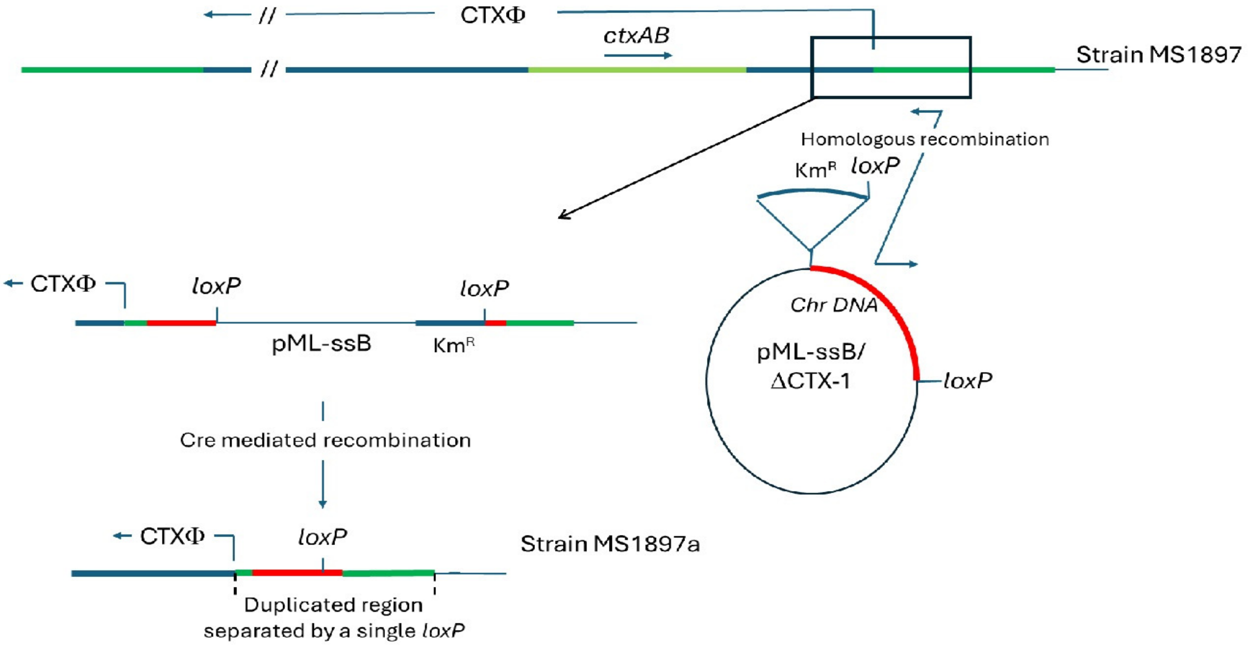
CTXΦ
ctxAB
Strain MS1897
Homologous recombination
Km^R loxP
Chr DNA
pML-ssB/
ΔCTX-1
loxP
CTXΦ
loxP
loxP
pML-ssB
Km^R
Cre mediated recombination
CTXΦ
loxP
Strain MS1897a
Duplicated region
separated by a single loxP

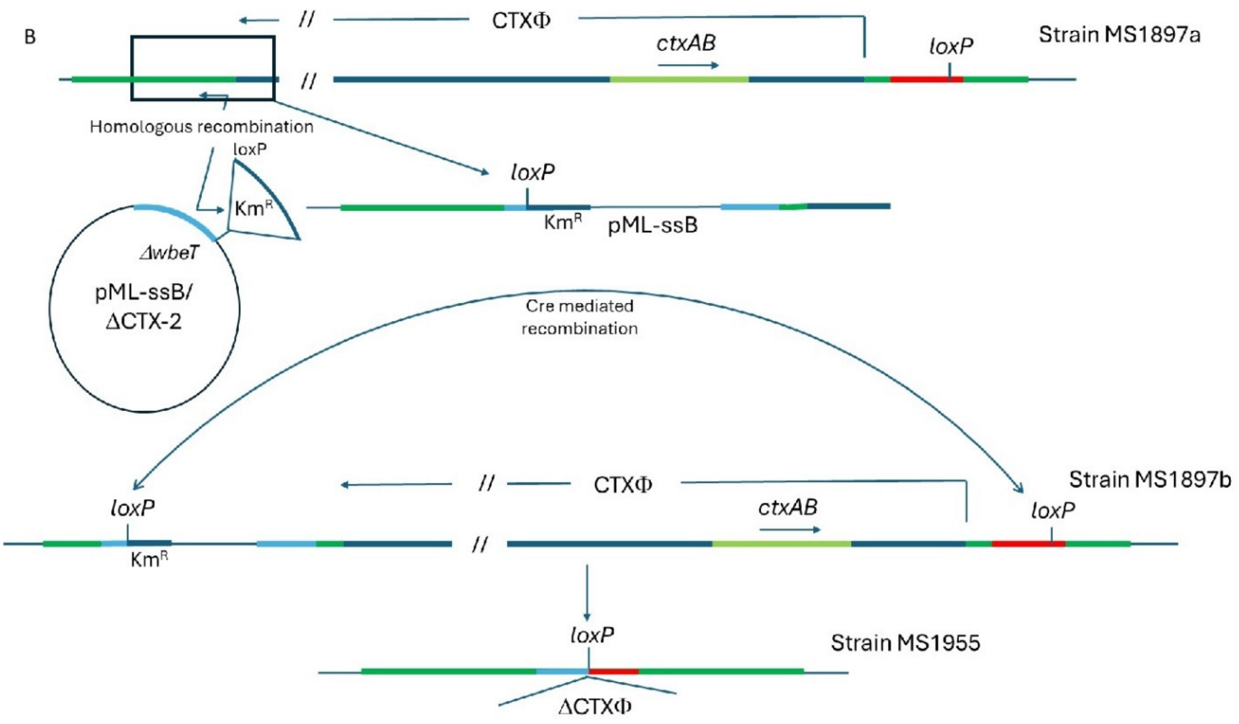

Figure S3 A. Insertion of a site downstream of the CTXΦ region in strain 1897. **B.** Insertion of a *loxP* site upstream of the the CTXΦ region and its removal by Cre-mediated recombination to produce strain MS1955.

1.3. Construction of MS1987

- **Reinsertion of the wild type wbeT gene into MS1955.**

Once strain MS1955 had been constructed and its genotype confirmed, the isogenic Ogawa derivative could be constructed by reinsertion of a wild type *wbeT* gene.

The *wbeT* gene and 500bp on either side of it were amplified by PCR from the El Tor Ogawa strain X25049. The primers introduced XbaI sites at either end of the fragment and these were used to clone the gene into the suicide vector pML-ssB (Figure 4A)).

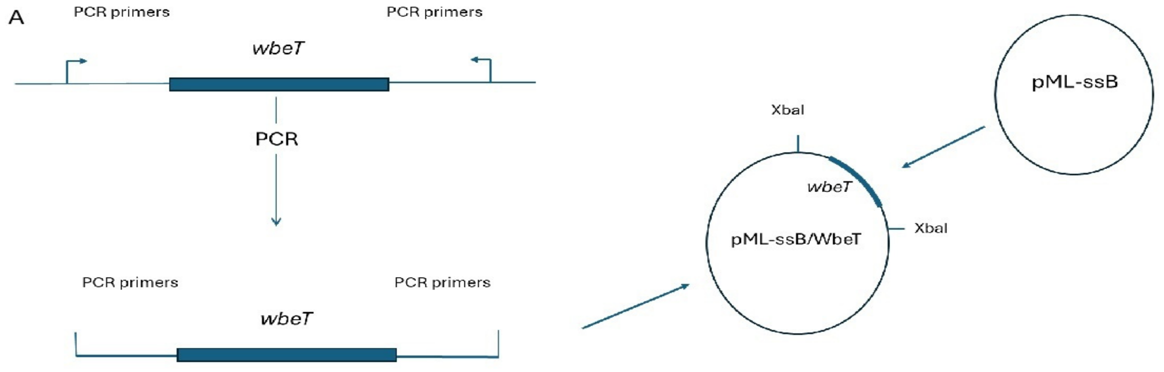

Figure S4A. Insertion of a *loxP* site upstream of the the CTXΦ region and its removal by Cre-mediated recombination to produce strain MS1955.

The resulting plasmid was transferred into strain MS1955 by transconjugation as described. Transconjugants were selected on the basis of chloramphenicol resistance. Transconjugants were confirmed to have acquired the Ogawa phenotype.

To remove the suicide vector from the chromosome transconjugants were subjected to sucrose selection as described. Strains were selected for loss of chloramphenicol resistance and retention of the Ogawa phenotype. The genotype was then confirmed by PCR analysis and sequencing. The final strain was **MS1987** (Figure 4B).

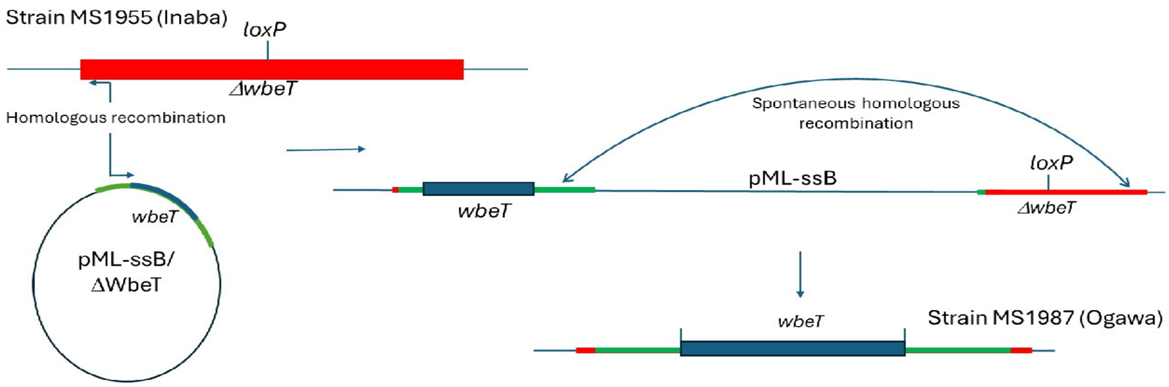

Figure S4B. Insertion of pML-ssB/wbeT into the chromosome of MS1955. The suicide vector was removed from the chromosome by spontaneous homologous recombination. Strains in which this occurred were selected using sucrose selection. Strains which had lost the plasmid

were resistant to sucrose. As described earlier, the presence of the *B. subtilis sacB* gene on the suicide vector, results in sucrose being lethal to cells retaining the plasmid.

Properties and stability of MM1955 and MS1987 strains

The strains have been used for more than 5 years in the Gotovax/UGOT labs and their genotype and phenotype properties have been extensively documented and proved to be highly stable:

1. They (and the parent Phil6973 strain) have been fully DNA sequenced and shown to have the expected sequences (data available on request)
2. They (and the parent Phil6973 strain) have been repeatedly analyzed by PCR methods for the presence and absence of the relevant modified genes with no change in results at years and many generations intervals – see **Appendix 2** for Methods description and photo illustration of results.
3. The serotype phenotype has been repeatedly tested at years and many generations intervals with the expected agglutination results.
4. Being isogenic these strains have had indistinguishable growth curves in media and this has been tested and proved to be stable over years and many generations intervals.

2. Vaccine strain MMS1692 producing rCTB component

2.1. Introduction.

The MMS1692 strain that is used for production of the rCTB component of Duochol was developed by Gotovax/UGOT as described in full below (and in a condensed form also in *Terrinoni, M., et al. Appl Environ Microbiol, 2017*). The strain is a classical biotype V. cholerae O1 Inaba strain. The strain is *ctxA* gene deleted and therefore cannot produce any cholera holotoxin, while it can still produce a low amount of CTB from its chromosomal *ctxB* gene. More than 99% of the CTB produced though is expressed constitutively from the *ctxB* gene under the control of the synthetic tac promoter on an inserted, high copy-number plasmid. The plasmid is maintained by complementation of the essential lgt gene with the corresponding gene from E. coli. The lgt gene has been deleted from the chromosome of the host strain which cannot survive without the plasmid. This provides a versatile strain for large-scale production of secreted CTB in media without any need for any antibiotic or specific ingredient (as needed for auxotrophic strains) for maintenance of the plasmid.

Origins of the vaccine strain.

The parent strain used for the construction of MMS1692 is JS1569, a rifampicin-resistant derivative of strain CVD103. CVD103 is a *ctxA*-deleted derivative of the wild-type clinical isolate 569B, classical biotype V. cholerae O1 Inaba, one of the most widely used isolates from the 6th pandemic.

CVD103 was constructed at the Center for Vaccine Development (CVD) at the Univ of Maryland, USA to create a safe strain for use as a live-attenuated oral cholera vaccine by removing the *ctxA* gene as described in Kaper JB et al. Nature. 1984;308(5960):655-8 & Kaper JB, Levine MM. Res Microbiol. 1990;141(7-8):901-6, and in condensed form below. To construct the attenuated V. cholerae strain CVD103, the cloned ctx genes were digested with the restriction enzymes XbaI and ClaI and a 550-bp fragment removed containing 94% of the sequences encoding the A1 peptide, the component responsible for the ADP-ribosylating toxic activity of the cholera enterotoxin. The remaining sequences were re-ligated and the resulting plasmid retained sequences encoding the A2 and B subunits as well as the promoter for the ctx operon. The mutated genes were introduced in a two-step procedure into the chromosome of the classical Inaba strain 569B. In the first step, an insertion mutation was made by cloning a gene encoding tetracycline resistance into the ctx gene which was then recombined into the chromosome of 569B. In the second step, the plasmid encoding the deletion

mutation was introduced into the tetracycline-resistant V. cholerae strain. Homologous recombination of the ctx deletion into the chromosome was detected by screening for tetracycline sensitivity. Both ctx gene copies in V. cholerae 569B were mutated in this manner and the resulting strain, CVDI03, could produce the B but not the A subunit of cholera enterotoxin (A-B+). The strain was shown to be safe and immunogenic in human volunteers. With the additional introduction of an *HgR* marker gene, this strain (CVD103-HgR) is the strain registered as the Vaxchora live-attenuated oral cholera vaccine.

JS1569 is a rifampicin resistant derivative of CVD103 and was used, as described by Sanchez J & Holmgren J (Proc Natl Acad Sci U S A. 1989 Jan;86(2):481-5) as the parent strain for harbouring a high-copy plasmid encoding for high-level production of CTB under control of the tac promoter, which latter strain is the strain used in Valneva´s rCTB production for the Dukoral oral cholera vaccine.

2.2. Construction of MMS1692.

NOTE: The strains and plasmids used in the work are listed in Table 1, and citation of "this study" in tables, figure legends or text refer to *Terrinoni, M., et al. Appl Environ Microbiol, 2017* as do the cited "reference numbers".

- ***Generation of a temperature-sensitive maintenance plasmid carrying native lgt from E.coli***

The *lgt* gene encoding a (pro)lipoprotein glyceryl transferase is essential for the biosynthesis of an essential bacterial lipoprotein. Mutations in *lgt* are lethal in V. cholerae and other gram negative organisms. The strategy for ensuring maintenance of the CTB encoding plasmid was to delete the *lgt* gene from the V.cholerae JS1569 strain and complement for this loss by the E.coli derived *lgt* gene provided in trans on a temperature sensitive plasmid allowing cells to grow at 30°C but not at 37°C.

In order to delete the *lgt* gene from the chromosome of V. cholerae, it must first be present in the strain on a plasmid to complement the loss of the native gene. Accordingly, the temperature-sensitive plasmid was constructed harboring the non-homologous *lgt* gene cloned from *E. coli*. A 1257 bp DNA fragment encoding the *lgt* gene was amplified from *E.coli* chromosomal template DNA using the primers lgt EC f and lgt EC r (**see figure S5 and table S1 for primer sequence**). This fragment was digested with HindIII and EcoRI and blunt end repaired with T4 DNA polymerase.

The temperature sensitive replicon derived from pSC101 was amplified from the plasmid pKD46 using primers pKD46f and pKD46r and also blunt end repaired with T4 DNA polymerase. The two fragments were then ligated together, and the ligated DNA was used to transform E. coli strain XL1 by electroporation. Ampicillin resistant transformants were then screened for plasmids carrying the *E.coli*-derived *lgt* encoding fragment using PCR and restriction analysis of purified plasmid DNA. All incubations following the electroporation of competent cells were done at 30°C. The resulting plasmid was pMT-lgtEc(ts) (see Figure 1 and Appendix 1, see Seq 1,) which was used to transform JS1569 (**figure S5**).

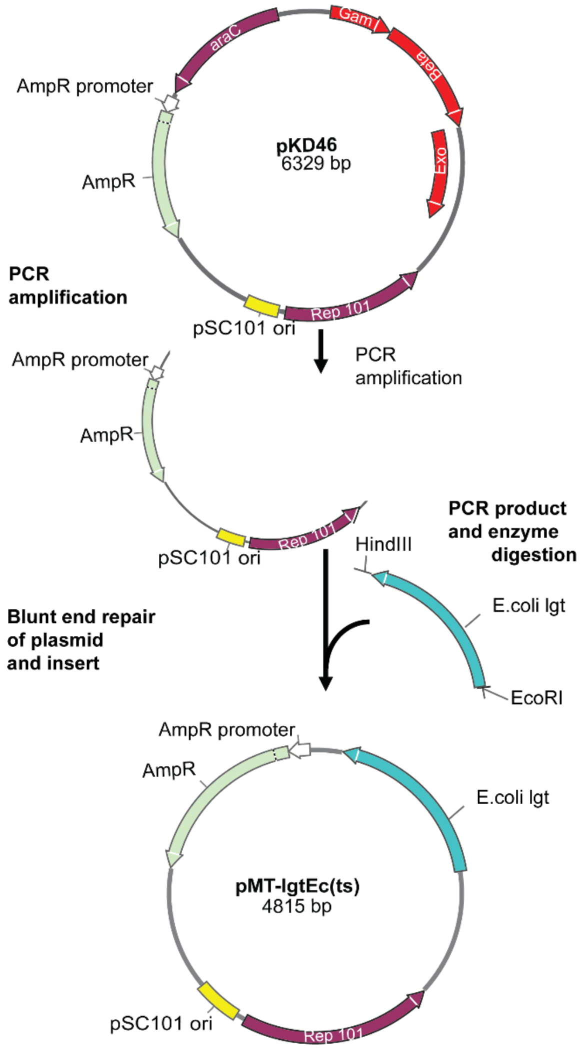

Figure S5. **Construction of the maintenance plasmid pMT- lgtEc(ts) plasmid.** pKD46 is derived from the temperature sensitive pSC101. The plasmid was amplified to obtain the replicon and ligated together with the *lgt*-encoding region amplified from *E. coli* with PCR primers lgt ECf and lgt ECr which resulted in a DNA fragment flanked by HindIII and EcoRI restriction sites. After enzyme digestion, the insert and plasmid were bluntend repaired and ligated. The ligated fragment was electroporated into electrocompetent V.cholerae JS1569.

- ***Generation of an lgt-deleted strain of V. cholerae .***

The entire sequence of the fragment from JS1569 encoding the *lgt* gene is shown in **SEQ2**. Two fragments were amplified from chromosomal template DNA obtained from *V. cholerae* using primer pairs VCD1/VCD2 and VCD3/VCD4 resulting in 334 bp and 573 bp fragments respectively (**SEQ3 and SEQ4 respectively**). These were fused together by primerless PCR using the primers VCD1 and VCD4 for amplification of the final 884 bp *lgt*-deleted fragment (**SEQ5**). The fragment was inserted into the pML-ssB suicide vector using EcoRV and BamHI. Ligated DNA was electroporated into *E. coli* strain S17-1 and transformants were selected on the basis of resistance to Cm. Plasmids were isolated from Cm resistant transformants and screened for insertion of the Δ*lgt* fragment by restriction analysis and PCR using the primers VCD1 and VCD4. The resulting plasmid was **pMT–ssB–ΔlgtVc.**

A DNA fragment carrying the Km resistance gene from Tn5 flanked by *FRT* sites was obtained from the plasmid pBC FRT/Km (constructed in this laboratory) by digestion with SalI and EcoRV. This fragment was blunt-end repaired and inserted into the *PvuII* site generated by the deletion in the *lgt* gene in pMT-ssB-ΔlgtVc. Ligated DNA was electroporated into *E. coli* strain S17-1 and transformants were selected by their resistance to both Cm and Km. Plasmids carried by clones with the correct antibiotic resistances were isolated and analyzed by PCR and restriction analysis of purified plasmid DNA. The resulting plasmid was pMT-ssB-ΔlgtVc/Km^R (**the insert is shown in appendix SEQ6 and figure S6).**

The plasmid pMT-ssB-ΔlgtVc/Km^R was then transferred into JS1569 carrying the temperature sensitive plasmid pMT-lgtEc(ts) by transconjugation on LB plates at 30˚C. Cells from these matings were grown on selective LB agar plates containing Amp and Km at 30˚C. Colonies obtained from the selective plates were then grown on M9 minimal medium agar containing Amp and Km in order to completely eliminate any cells from the donor S17-1 strain. The resulting strain resistant to kanamycin, ampicillin and chloramphenicol was grown up in 5mL liquid LB broth supplemented Km and Amp only at 30˚C overnight and then streaked out onto LB-agar plates containing no salt but supplemented with sucrose to a final concentration of 6%. Single colonies from the sucrose plate were isolated and patched onto LB-agar plates supplemented with Km and Amp and checked for sensitivity to Cm by duplicate plating onto LB-agar plates containing Cm. Cm sensitive colonies were confirmed to be *V. cholerae* derivatives by PCR confirming the absence of the R6K *pir* gene present

only in the S17-1 donor. The absence of the chromosomal *lgt* gene in the strains and its replacement with the Km resistance gene were both confirmed by PCR using primers VCD1 and VCD4 followed by DNA sequencing. The resulting strain was called MMS1588 [JS1569 *ΔlgtKm*^{R}(Tn5),*pMT/lgtEc(ts)*].

The Km resistance gene was removed from strain MMS1588 by FLP-mediated recombination. However, the commercially available plasmid for this procedure was based on the same replicon as pMT-lgtEc(ts) and could not be used. We therefore constructed a plasmid (pMT-FRT) based on the pBR322 origin of replication in which the *FRT* gene was expressed from the *tac* promoter under the control of the *lacIq* repressor. Furthermore, since pMT-lgtEc(ts) carries the *bla* gene conferring Amp resistance, we used the *cat* gene to confer Cm resistance. Thus, cells of MMS1588 were transformed with the pMT-FRT plasmid and selected on the basis of Cm resistance. Transformants were streaked out on LB-agar plates supplemented with Amp, Cm and 1mM IPTG (for induction of FRT recombinase expression) and

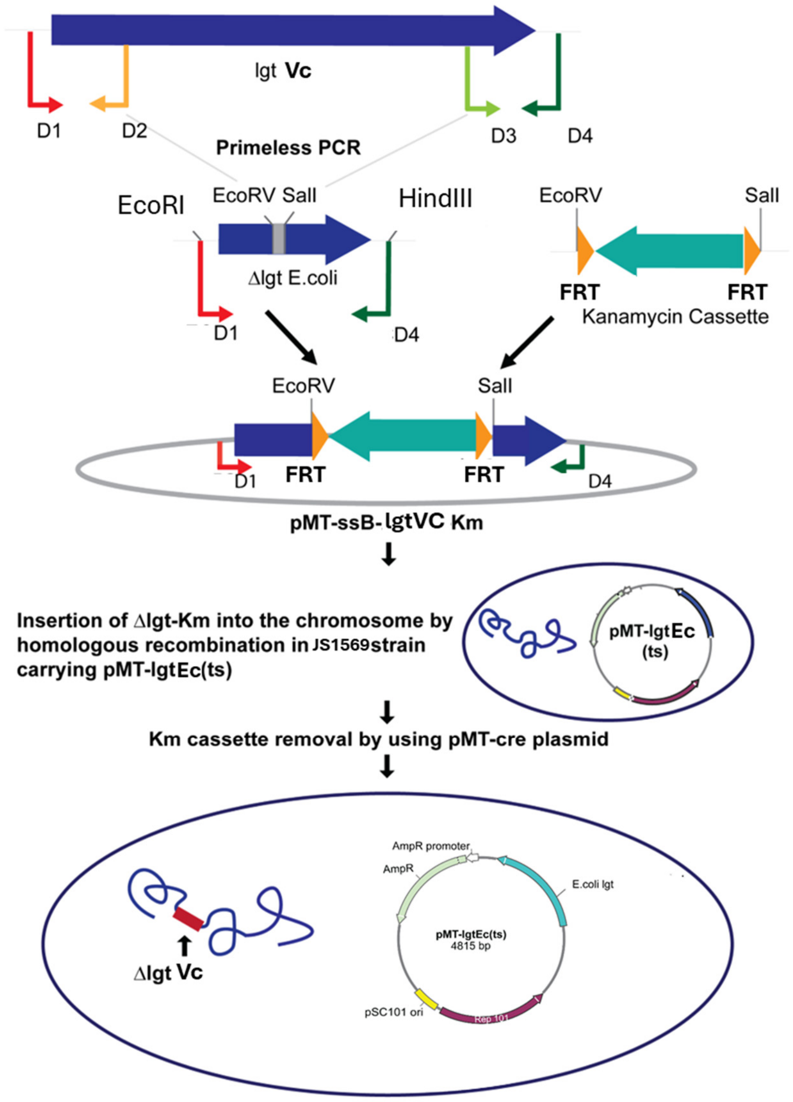

.

Figure S6. Schematic representation of the construction of Δlgt strains of V. cholerae carrying temperature sensitive plasmid pMT-lgtEc(ts). DNA fragments carrying a deletion of lgt were generated by primerlessPCR and cloned into suicide plasmid pMT-ssB. A kanamycin resistance gene flanked by loxP sites was then inserted in place of the deleted lgt gene. The resulting plasmids were used to create deletions in the chromosomes of V.cholerae by allelic exchange. The kanamycin resistance gene was then removed with Cre recombinase. A temperature-sensitive (ts) plasmid carrying a nonhomologous lgt gene complements the chromosomal deletion, allowing the survival of the strain at 30°C.

incubated at 30˚C. Clones were patched in duplicate onto LB-agar plates supplemented with either both Amp and Cm or with Km alone and incubated at 30˚C. Km sensitive colonies were then streaked out on LB-agar plates supplemented with Amp and grown at 30˚C overnight in order to allow the inherently unstable pMT-Cre plasmid to segregate out in the absence of selection. Single colonies were patched in

duplicate onto LB-agar plates supplemented with Amp or Cm and grown at 30°C. Amp resistant colonies that were sensitive to both Km and Cm were analyzed by PCR amplification of chromosomal DNA with primers VCD1 and VCD4 and DNA sequencing to ensure that the Km resistance gene was removed (Figure S6, **see SEQ7**). The DNA was also amplified with primers lgtEcf and lgtEcr in order to confirm the presence of the complementing *lgt* gene from *V. cholerae*.
The final strain was MMS1633 [JS1569: *Δlgt, pMT/lgtEC(ts)*] see **figure S6.**

- ***Construction of the rCTB vector, and its transformation into the MMS1633 strain.***

A feature of the system described is its use for the expression of recombinant rCTB without the need for antibiotic selection for the maintenance of plasmid expression vectors. This was achieved using the temperature-sensitive nature of the complementation maintenance vector.

The pML-LCTBtac expression plasmid carrying *ctxB* and derived from pAFtac1 was used as a starting plasmid. In order to obtain constitutive expression the lacIq gene was removed by digestion with BamHI and BglII and relegation. To raise the copy number of the plasmid the origin of replication from pBR322 was replaced by that from pUC19 and finally, the ampicillin resistance gene was replaced by the lgt gene derived from *E. coli.* The resulting fragments were digested with BspHI. The *lgt* gene was then amplified from *BL21 E.coli* using primers BspHIlgtEC f and BspHIlgtEC r that resulted in an amplified fragment flanked with BspHI sites. This fragment was also digested with BspHI and ligated together with the amplified plasmid fragments from the expression vector.

Strain MMS1633 was maintained at 30°C and made electrocompetent. The ligated DNA from the expression vector constructions was electroporated into the competent cells which were, following expression at 30°C, plated out on LB-agar plates without any antibiotics and incubated at 39°C. At this non-permissive temperature, the original strain cannot survive. Strains that had acquired the temperature-insensitive expression vector however would survive since the plasmids are retained at the higher temperature. Single colonies were picked from the transformation plates and tested for their sensitivity to Amp resulting from the loss of the temperature sensitive plasmid. The presence of the expression vector in the resulting strains was confirmed by restriction analysis of isolated plasmids. The chromosomal deletion of the native

lgt gene was also confirmed by PCR. The resulting plasmid was pMT-CTB/lgEC in **figure S7** and the strategy for introduction of the expression vector is shown in **figure S8.**

The resulting strain is called MMS1692 (MMS1633 carrying CTB expression plasmid pMT-CTB/lgtEc in place of pMT-lgtEc(ts)).

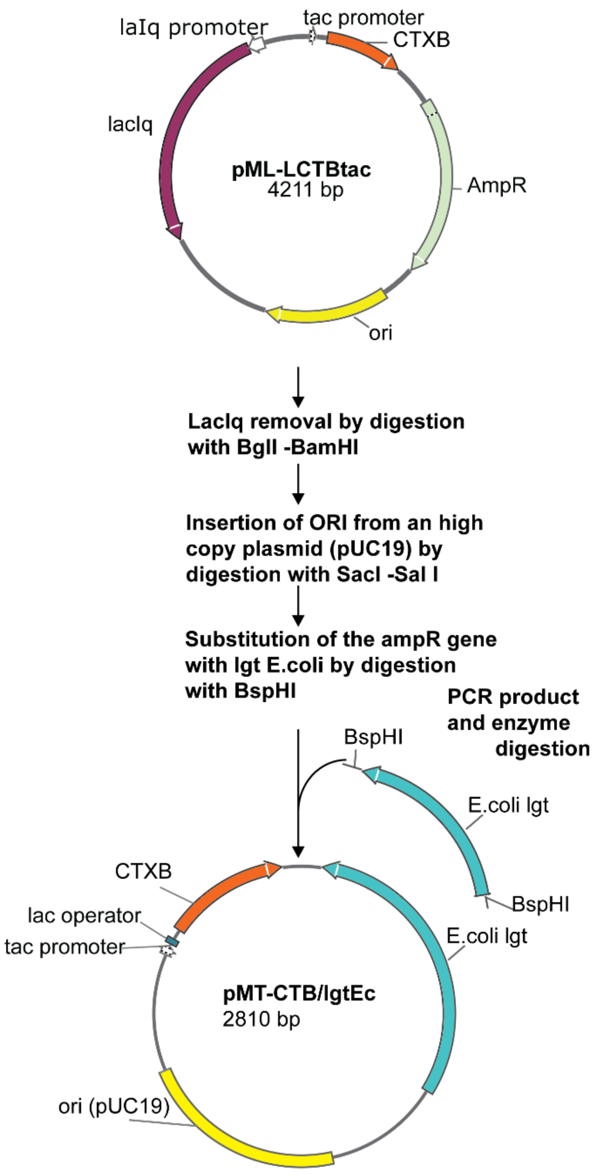

Figure S7. Summary of the construction of the CTB production plasmid pMT-CTB/lgtEc. The pML-LCTBtac expression plasmid carrying *ctxB* derived from pAFtac1 was used as a

starting plasmid. To obtain constitutive expression the lacIq gene was removed by digestion with BamHI and BglII and relegation. To raise the copy number of the plasmid the origin of replication from pBR322 was replaced by that from pUC19 and finally, the ampicillin resistance gene was replaced by the lgt gene derived from *E. coli*.

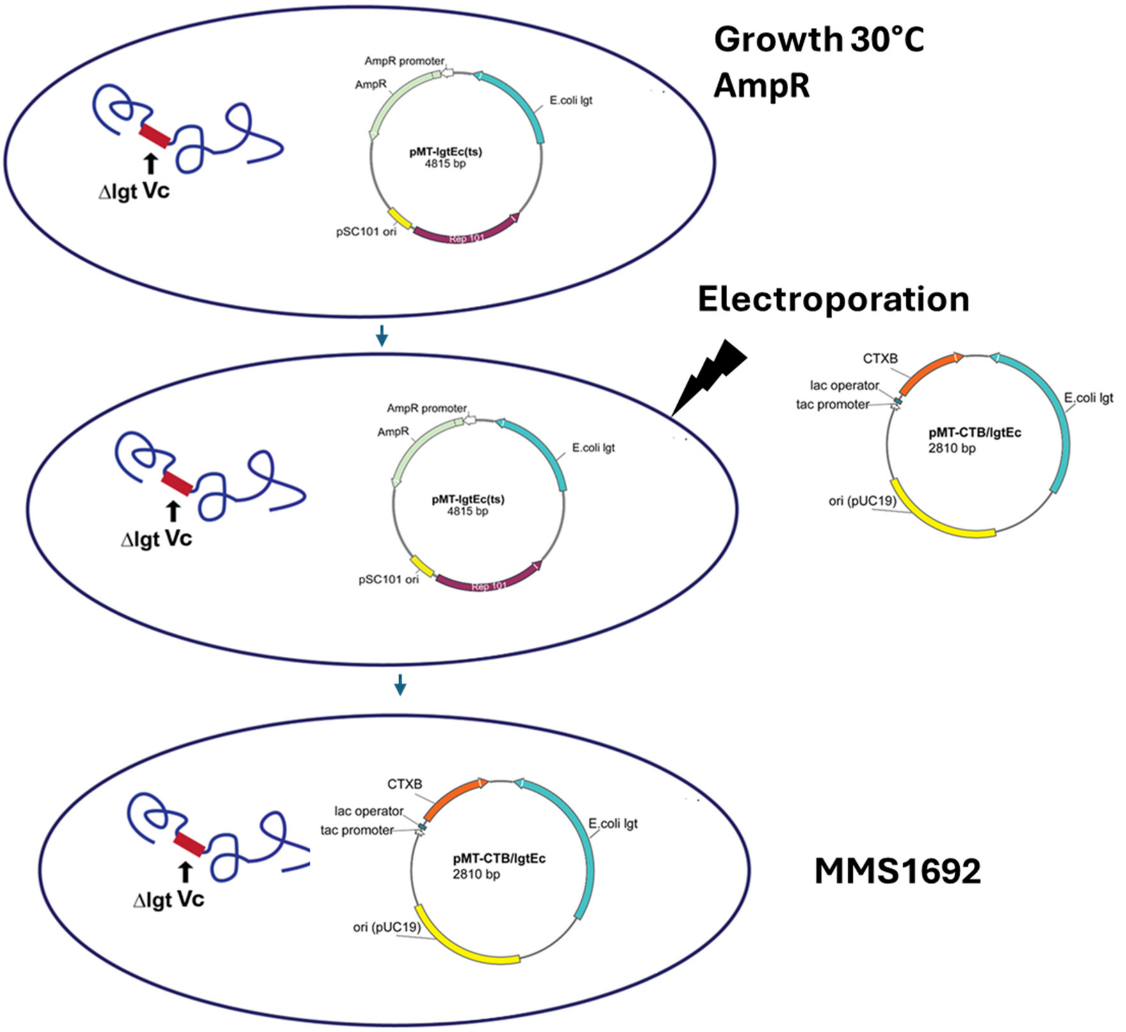

Figure S8. Replacement of the temperature-sensitive maintenance plasmid pMT-lgtEC(ts) in E. coli strain MMS1633 with an pMT-CTB/lgtEc for production of recombinant cholera B subunit (rCTB). Cells are made electrocompetent and electroporated with a temperature-insensitive plasmid that can replicate at 39°C. The cells are plated out onto LB agar plates containing no antibiotics and incubated at the higher temperature. Colonies are tested for sensitivity to Amp. The resulting strain carrying the expression rCTB vector is MMS1692.

2.3. Properties and stability of MMS1692

The MMS1692 strain has been used for more than 8 years in the Gotovax/UGOT laboratories and its genotype and phenotype properties have been extensively documented and proved to be stable:

1. The strain has been fully DNA sequenced and shown to have the expected chromosomal as well as plasmid sequences (data available on request).

2. The strain has been repeatedly analyzed by PCR methods for the presence and absence of the relevant modified genes with no change in results over years and many generations intervals – see **Appendix 2** for Methods description and photo illustration of results.

3. The serotype has been repeatedly tested over many years and generations with the expected seroagglutination results.

As described in *Terrinoni, M., et al. Appl Environ Microbiol, 2017*, the production of CTB protein from MMS1692 was compared with that from our before strongest rCTB-overproducing auxotrophic strain MS1012 (also derived from JS1569), in which the CTB-expressing plasmid is maintained by complementation of the deleted thyA gene by added thymine to the substrate. GM1-ELISA, which is based on receptor-specific binding of protein to surface-coated GM1 ganglioside followed by detection of the bound protein by a B-subunit specific monoclonal antibody, was used to detect and quantify CTB. The results demonstrated that the rCTB protein was produced and secreted in pentameric form in similar quantity from MMS1692 as from MS1012, 1.0 gram per Liter fermentation medium, and bound to GM1 and reacted with the monoclonal antibody in an identical manner.

FURTHER MMS1692 DETAILS

Table S1. Strains and plasmids used in the construction of MMS1692

Strain	Pheno/genotype	Source
Vibrio cholerae		
**MS1569**	V.c. JS1569. Classical, Inaba strain	
**MMS1588**	V.c. JS1569 pMT–suicide1–ΔlgtVC-Km carrying the temperature sensitive pMM-lgtEC(ts) plasmid.	This study
**MMS1589**	Δ*lgt* Tn5(km^R) derivative of JS1569 carrying the temperature sensitive pMM-lgtEC(ts) plasmid.	This Study

**MMS1633**	MMS1589 in which the Km^R gene has been removed.	This study
**MMS1692**	MMS1633 carrying pMM-CTB/lgtEC plasmid in place of pMM-lgtEC(ts)	This study
Plasmids		
**pAFtac-1**	Expression vector for cloning and expression of recombinant proteins from the *tac* promoter, confers ampicillin resistance	(24)
**pMT-ssB**	R6K-based suicide vector carrying the *sacB* gene from B. subtilis.	(3, 4)
**pMT–ssB–ΔlgtVc**	pMT-suicide1 carrying the deleted lgt *V. cholerae* gene (ΔlgtVc)	This study
**pMT-ssB-ΔlgtVC/Km^R**	pMT- suicide1 carrying the deleted lgt *V. cholerae* gene (ΔlgtVc) and Kanamycin gene (Km^R).	This study
**pBC FRT/Km**	Used as a source of the *FRT*-flanked Km^R gene. Confers Cm^R and Km^R	This study
**pMT-lgtEc(ts)**	pSC101-derived temperature sensitive plasmid carrying *E. coli* derived *lgt* gene	This study
**pMT-FRT**	pBR322-derived plasmid encoding the FLP recombinase expressed from the *tac* promoter under the control of the *lacI*^{q} repressor.	This study
**pMT-rCTB/lgtEC**	pMT plasmid carrying rCTB and *E. coli*-derived *lgt* gene.	This study

Table S2. Primers used and their sequences

Primer name	Sequence
**VCD1**	5′-GCGATTGCGATATCGGGCATGATTGG-3′
**VCD2**	5′-GCGCACCGCTAGAGGGCCGATCG-3′
**VCD3**	5′CGATCGGCCCTCTAGCGGTGCGCAGCTGTCGCGGTTTGTATCAAGACCGTGTAG-3′
**VCD4**	5′-GGGGGATCCGGCTGTACATGCAAGGGCGCAAACACC-3′
**lgt EC f**	5′- GGGGGGTCTCGAATTCGCTGCGGCGCATTGATTATGCCGAAG -3′
**lgtVC r**	5′-GGGGGGTCTCAAGCTTCTGTGTGCCTTCGTCGAGCACTTTTTGC -3′

Sequence data from the construction of the *V. cholerae* strain JS1569 *lgt*-deleted derivative MMS1663

SEQ 1- *lgt* gene amplified from *Escherichia coli* using primers lgt ECf and lgt ECr (underlined). The coding region is highlighted in blue. The PCR product was digested with EcoRI and HindIII, blunt-end repaired and inserted into temperature-sensitive vector derived from pSC101 (see cloning strategy in **Figure 1**):

1 GGGGGGTCTC GAATTCGCTG CGGCGCATTG ATTATGCCGA AGCAGAAAAT CTTGCGCAGC
61 GTAGTCTGGA AGCGCAACTG GCGACCGAAG TTCGCCATCA GGTTGCAGCC TTTATGGAGC
121 GTCGCGGCAT GGGCGGGCTG ATTCGCGGAG GGTTATAGCG CGGATCATAT ACATATCTTT
181 TAACGGTATC CGGCAACCAG CCAGGTCCCC TTGTGCTATT ATTCGCACCT TTGGAGCGCC
241 TGAAACCTGC GGCGCGCATT TCAATCGCTG TTCTCTTTCA GCGAAATAAC AAGAACTTGT
301 GGTGACAG**AT GACCAGTAGC TATCTGCATT TTCCGGAGTT TGATCCGGTC ATTTTCTCAA**
361 TAGGACCCGT GGCGCTTCAC TGGTACGGCC TGATGTATCT GGTGGGTTTC ATTTTTGCAA
421 TGTGGCTGGC AACACGACGG GCGAATCGTC CGGGCAGCGG CTGGACCAAA AATGAAGTTG
481 AAAACTTACT CTATGCGGGC TTCCTCGGCG TCTTCCTCGG GGGACGTATT GGTTATGTTC
541 TGTTCTACAA TTTCCCGCAG TTTATGGCCG ATCCGCTGTA TCTGTTCCGT GTCTGGGACG
601 GCGGCATGTC TTTCCACGGC GGCCTGATTG GCGTTATCGT GGTGATGATT ATCTTCGCCC
661 GCCGTACTAA ACGTTCCTTC TTCCAGGTCT CTGATTTTAT CGCACCACTC ATTCCGTTTG
721 GTCTTGGTGC CGGGCGTCTG GGCAACTTTA TTAACGGTGA ATTGTGGGGC CGCGTTGACC
781 CGAACTTCCC GTTTGCCATG CTGTTCCCTG GCTCCCGTAC AGAAGATATT TTGCTGCTGC
841 AAACCAACCC GCAGTGGCAA TCCATTTTCG ACACTTACGG TGTGCTGCCG CGCCACCCAT
901 CACAGCTTTA CGAGCTGCTG CTGGAAGGTG TGGTGCTGTT TATTATCCTC AACCTGTATA
961 TTCGTAAACC ACGCCCAATG GGAGCTGTCT CAGGTTTGTT CCTGATTGGT TACGGCGCGT
1021 TTCGCATCAT TGTTGAGTTT TTCCGCCAGC CCGACGCGCA GTTTACCGGT GCCTGGGTGC
1081 AGTACATCAG CATGGGGCAA ATTCTTTCCA TCCCGATGAT TGTCGCGGGT GTGATCATGA
1141 TGGTCTGGGC ATATCGTCGC AGCCCACAGC AACACGTTTC CTGAGGAACC ATGAAACAGT
1201 ATTTAGAACT GATGCAAAAA GTGCTCGACG AAGGCACACA GAAGCTTGAG ACCCCCC

SEQ 2- Region of *V. cholerae* chromosome encoding *lgt* (coding region underlined) with the deleted region highlighted in blue:

1 GCGGTACCGG CATTGGGGTA ATCTCAAGTT TGGCCGGTAT TGGCGGCGGT TCTTTATCGG
61 TGCCATTTTT GAATCGACAT GGCATTGAAA TGAAAAAGGC GATAGGTTCT TCATCGGTCT
121 GTGGTTTTGC GATTGCGATA TCGGGCATGA TTGGTTTTAT TTTGCACGGT TATCAAGTGG
181 AGAACTTGCC ACAATACAGC CTTGGTTATG TTTATTTACC TGCATTGTTA GCGATTGCTA
241 CAACATCGAT GCTTACCACG CGAATTGGCG CTAAACTTGC CACCCAAATG CCAACAGCAA
301 GGCTTAAGCG ATTCTTTGCC ATTTTTTTAA TGTGCGTCGC TGTGACCATG TTGTTCCAGT
361 AATACTCATT GTTTATAGAG AAGGTTTGTT ATGCCTCAGG GTTATCTGCA GTTTCCCAAT
421 ATTGACCCCG TATTGTTTTC GATCGGCCCT CTAGCGGTGC GCTGGTATGG CTTGATGTAT
481 TTGGTGGGTT TCCTTTTTGC TATGTGGTTG GCCAATCGCC GAGCGGATCG CGCGGGCAGT
541 GGTTGGACGC GTGAGCAAGT CTCTGACTTG TTATTCGCCG GCTTTTTAGG TGTAGTGATC
601 GGTGGCCGAG TTGGTTATGT GATCTTCTAC AATTTTGATC TGTTCCTTGC TGACCCTCTT
661 TATTTATTCA AAGTGTGGAC TGGCGGCATG TCCTTCCACG GCGGCTTATT GGGTGTGATC
721 ACCGCCATGT TCTGGTATGC GCGTAAAAAC CAACGCACCT TCTTTGGTGT GGCCGATTTT
781 GTTGCCCCTT TAGTGCCATT CGGTTTGGGG ATGGGACGTA TCGGTAACTT TATGAATAGT
841 GAACTTTGGG GACGAGTAAC GGATGTGCCT TGGGCTTTTG TATTCCCTAA TGGTGGCCCA
901 CTGCCGCGCC ATCCTTCACA GCTTTATGAA TTCGCCTTAG AAGGCGTGGT GCGTATCCGG
961 TCTGTTCTTT ATTCTTAATT GGTTTATTGG TAAACCTCGT CCGCTAGGCA ACTGTTTTTA
1021 GCTGGATACG GTACATTCCG CTTCCTTGTG GAATACGTCC GTGAGCCAGA TGCTCAGTTG
1081 GGTCTGTTTG GTGGCTTCAT TTCAATGGGG CAAATCCTCT CCTTACCTAT GGTGATCATC
1141 GGTATTTTGA TGATGGTTTG GTCTTACAAG CGCGGTTTGT ATCAAGACCG TGTAGCAGCA
1201 AAATAGGGTA GTTAGGTGAG ACAGTATTTA GATCTTTGTC AGCGCATCGT CGATCAAGGT
1261 GTTTGGGTTG AAAATGAACG AACGGGCAAG CGTTGTTTGA CTGTGATTAA TGCCGATTTG
1321 ACCTACGATG TGGGCAACAA TCAGTTTCCT CTAGTGACTA CACGCAAGAG TTTTTGGAAA
1381 GCTGCCGTAG CCGAGTTGCT CGGCTATATT CGTGGTTACG ATAATGCGGC GGATTTTCGC
1441 CAATTAGGTA CCAAAACCTG GGATGCTAAT GCCAATTTAA ACCAAGCATG GCTCAACAAT
1501 CCTTACCGTA AAGGTGAGGA TGACATGGGA CGCGTGTATG GTGTTCAGGG TAGAGCTTGG
1561 GCTAAGCCTG ATGGTGGTCA TATTGACCAG TTGAAAAAGA TTGTTGATGA TTTGAGCCGT
1621 GGCGTTGATG ACCGAGGTGA AATTCTTAAC TTCTACAATC CGGGTGAATT TCACATGGGG
1681 TGTTTGCGCC CTTGCATGTA CAGCCATCAT TTTTCATTGC TGGGGGATAC CTTGTATCTC
1741 AACAGTACTC AGCGTTCATG TGATGTGCCC TTGGGGTTGA ATTTCAACAT GGTGCAGGTT
1801 TATGTGTTCC TTGCGCTGAT GG

SEQ3 - Amplified DNA using primer pair VCD1/ VCD2 (Primer sequences underlined):

1 GCGATTGCGA TATCGGGCAT GATTGGTTTT ATTTTGCACG GTTATCAAGT GGAGAACTTG
51 CCACAATACA GCCTTGGTTA TGTTTATTTA CCTGCATTGT TAGCGATTGC TACAACATCG
101 ATGCTTACCA CGCGAATTGG CGCTAAACTT GCCACCCAAA TGCCAACAGC AAGGCTTAAG
151 CGATTCTTTG CCATTTTTTT AATGTGCGTC GCTGTGACCA TGTTGTTCCA GTAATACTCA
201 TTGTTTATAG AGAAGGTTTG TTATGCCTCA GGGTTATCTG CAGTTTCCCA ATATTGACCC
251 CGTATTGTTT TCGATCGGCC CTCTAGCGGT GCGC

SEQ 4 - Amplified DNA using primer pair VCD3/ VCD4 (primer sequences underlined) The PvuII site replacing the *lgt* gene is highlighted:

1 CGATCGGCCC TCTAGCGGTG CGCAGCTGTC GCGGTTTGTA TCAAGACCGT GTAGCAGCAA
61 AATAGGGTAG TTAGGTGAGA CAGTATTTAG ATCTTTGTCA GCGCATCGTC GATCAAGGTG
121 TTTGGGTTGA AAATGAACGA ACGGGCAAGC GTTGTTTGAC TGTGATTAAT GCCGATTTGA
181 CCTACGATGT GGGCAACAAT CAGTTTCCTC TAGTGACTAC ACGCAAGAGT TTTTGGAAAG
241 CTGCCGTAGC CGAGTTGCTC GGCTATATTC GTGGTTACGA TAATGCGGCG GATTTTCGCC
301 AATTAGGTAC CAAAACCTGG GATGCTAATG CCAATTTAAA CCAAGCATGG CTCAACAATC
361 CTTACCGTAA AGGTGAGGAT GACATGGGAC GCGTGTATGG TGTTCAGGGT AGAGCTTGGG
421 CTAAGCCTGA TGGTGGTCAT ATTGACCAGT TGAAAAAGAT TGTTGATGAT TTGAGCCGTG
481 GCGTTGATGA CCGAGGTGAA ATTCTTAACT TCTACAATCC GGGTGAATTT CACATGGGGT
541 GTTTGCGCCC TTGCATGTAC AGCCGGATCC CCC

SEQ 5 - Amplified DNA following primerless PCR and amplification with VCD1 and VCD4. A unique PvuII (highlighted in blue) site replaces the *lgt* gene sequence:

1 GCGATTGCGA TATCGGGCAT GATTGGTTTT ATTTTGCACG GTTATCAAGT GGAGAACTTG
61 CCACAATACA GCCTTGGTTA TGTTTATTTA CCTGCATTGT TAGCGATTGC TACAACATCG
121 ATGCTTACCA CGCGAATTGG CGCTAAACTT GCCACCCAAA TGCCAACAGC AAGGCTTAAG
181 CGATTCTTTG CCATTTTTTT AATGTGCGTC GCTGTGACCA TGTTGTTCCA GTAATACTCA
241 TTGTTTATAG AGAAGGTTTG TTATGCCTCA GGGTTATCTG CAGTTTCCCA ATATTGACCC
301 CGTATTGTTT TCGATCGGCC CTCTAGCGGT GCGCAGCTG CGCGGTTTGT ATCAAGACCG
361 TGTAGCAGCA AAATAGGGTA GTTAGGTGAG ACAGTATTTA GATCTTTGTC AGCGCATCGT
421 CGATCAAGGT GTTTGGGTTG AAAATGAACG AACGGGCAAG CGTTGTTTGA CTGTGATTAA
481 TGCCGATTTG ACCTACGATG TGGGCAACAA TCAGTTTCCT CTAGTGACTA CACGCAAGAG
541 TTTTTGGAAA GCTGCCGTAG CCGAGTTGCT CGGCTATATT CGTGGTTACG ATAATGCGGC
601 GGATTTTCGC CAATTAGGTA CCAAAACCTG GGATGCTAAT GCCAATTTAA ACCAAGCATG
661 GCTCAACAAT CCTTACCGTA AAGGTGAGGA TGACATGGGA CGCGTGTATG GTGTTCAGGG
721 TAGAGCTTGG GCTAAGCCTG ATGGTGGTCA TATTGACCAG TTGAAAAAGA TTGTTGATGA
781 TTTGAGCCGT GGCGTTGATG ACCGAGGTGA AATTCTTAAC TTCTACAATC CGGGTGAATT
841 TCACATGGGG TGTTTGCGCC CTTGCATGTA CAGCCGGATC CCCC

The fragment was inserted into the pML-ssB suicide vector using EcoRV and BamHI. The EcoRV/SalI fragment carrying Kanamycin gene the flanked by FRT sites was blunt-end repaired and inserted into the PvuII site.

SEQ 6- DNA fragment carrying the kanamycin resistance cassette and FRT sites (highlighted) The kanamycin resistance gene is underlined:

1 GCGATTGCGA TATCGGGCAT GATTGGTTTT ATTTTGCACG GTTATCAAGT
51 GGAGAACTTG CCACAATACA GCCTTGGTTA TGTTTATTTA CCTGCATTGT
101 TAGCGATTGC TACAACATCG ATGCTTACCA CGCGAATTGG CGCTAAACTT

151 GCCACCCAAA TGCCAACAGC AAGGCTTAAG CGATTCTTTG CCATTTTTTT
201 AATGTGCGTC GCTGTGACCA TGTTGTTCCA GTAATACTCA TTGTTTATAG
251 AGAAGGTTTG TTATGCCTCA GGGTTATCTG CAGTTTCCCA ATATTGACCC
301 CGTATTGTTT TCGATCGGCC CTCTAGCGGT GCGCAGATCA GAAGTTCCTA
351 TTCTCTAGAA AGTATAGGAA CTTCGCGGCC GGGGGCGCTG AGGTCTGCCT
401 CGTGAAGAAG GTGTTGCTGA CTCATACCAG GCCTGAATCG CCCCATCATC
451 CAGCCAGAAA GTGAGGGAGC CACGGTTGAT GAGAGCTTTG TTGTAGGTGG
501 ACCAGTTGGT GATTTTGAAC TTTTGCTTTG CCACGGAACG GTCTGCGTTG
551 TCGGGAAGAT GCGTGATCTG ATCCTTCAAC TCAGCAAAAG TTCGATTTAT
601 TCAACAAAGC CGCCGTCCCG TCAAGTCAGC GTAATGCTCT GCCAGTGTTA
651 CAACCAATTA ACCAATTCTG ATTAGAAAAA CTCATCGAGC ATCAAATGAA
701 ACTGCAATTT ATTCATATCA GGATTATCAA TACCATATTT TTGAAAAAGC
751 CGTTTCTGTA ATGAAGGAGA AAACTCACCG AGGCAGTTCC ATAGGATGGC
801 AAGATCCTGG TATCGGTCTG CGATTCCGAC TCGTCCAACA TCAATACAAC
851 CTATTAATTT CCCCTCGTCA AAAATAAGGT TATCAAGTGA GAAATCACCA
901 TGAGTGACGA CTGAATCCGG TGAGAATGGC AAGAGCTTAT GCATTTCTTT
951 CCAGACTTGT TCAACAGGCC AGCCATTACG CTCGTCATCA AAATCACTCG
1001 CATCAACCAA ACCGTTATTC ATTCGTGATT GCGCCTGAGC GAGACGAAAT
1051 ACGCGATCGC TGTTAAAAGG ACAATTACAA ACAGGAATCG AATGCAACCG
1101 GCGCAGGAAC ACTGCCAGCG CATCAACAAT ATTTTCACCT GAATCAGGAT
1151 ATTCTTCTAA TACCTGGAAT GCTGTTTTCC CGGGGATCGC AGTGGTGAGT
1201 AACCATGCAT CATCAGGAGT ACGGATAAAA TGCTTGATGG TCGGAAGAGG
1251 CATAAATTCC GTCAGCCAGT TTAGTCTGAC CATCTCATCT GTAACATCAT
1301 TGGCAACGCT ACCTTTGCCA TGTTTCAGAA ACAACTCTGG CGCATCGGGC
1351 TTCCCATACA ATCGATAGAT TGTCGCACCT GATTGCCCGA CATTATCGCG
1401 AGCCCATTTA TACCCATATA AATCAGCATC CATGTTGGAA TTTAATCGCG
1451 GCCTCGAGCA AGACGTTTCC CGTTGAATAT GGCTCATAAC ACCCCTTGTA
1501 TTACTGTTTA TGTAAGCAGA CAGTTTTATT GTTCATGATG ATATATTTTT
1551 ATCTTGTGCA ATGTAACATC AGAGATTTTG AGACACAACG TGGCTTTCCC
1601 GGCCGCCCGT GGCAGAAGTT CCTATTCTCT AGAAAGTATA GGAACTTCAG
1651 TCGACTGTCG CGGTTTGTAT CAAGACCGTG TAGCAGCAAA ATAGGGTAGT
1701 TAGGTGAGAC AGTATTTAGA TCTTTGTCAG CGCATCGTCG ATCAAGGTGT
1751 TTGGGTTGAA AATGAACGAA CGGGCAAGCG TTGTTTGACT GTGATTAATG
1801 CCGATTTGAC CTACGATGTG GGCAACAATC AGTTTCCTCT AGTGACTACA
1851 CGCAAGAGTT TTTGGAAAGC TGCCGTAGCC GAGTTGCTCG GCTATATTCG
1901 TGGTTACGAT AATGCGGCGG ATTTTCGCCA ATTAGGTACC AAAACCTGGG
1951 ATGCTAATGC CAATTTAAAC CAAGCATGGC TCAACAATCC TTACCGTAAA
2001 GGTGAGGATG ACATGGGACG CGTGTATGGT GTTCAGGGTA GAGCTTGGGC
2051 TAAGCCTGAT GGTGGTCATA TTGACCAGTT GAAAAAGATT GTTGATGATT
2101 TGAGCCGTGG CGTTGATGAC CGAGGTGAAA TTCTTAACTT CTACAATCCG
2151 GGTGAATTTC ACATGGGGTG TTTGCGCCCT TGCATGTACA GCCGGATCCC
2201 CC

SEQ 7 - Chromosomal fragment from MMS1663 in which the kanamycin resistance cassette has been removed by FLP-mediated recombination leaving a single FRT site (highlighted in blue). The whole genome sequence shown below shows that the recombination excision was imprecise but nonetheless resulted in the deletion of the

lgt gene and the retention of a single FRT site. The red sequence shows the DNA replacing the original *lgt* sequence:

1 GCGGTACCGG CATTGGGGTA ATCTCAAGTT TGGCCGGTAT TGGCGGCGGT
51 TCTTTATCGG TGCCATTTTT GAATCGACAT GGCATTGAAA TGAAAAAGGC
101 GATAGGTTCT TCATCGGTCT GTGGTTTTGC GATTGCGATA TCGGGCATGA
151 TTGGTTTTAT TTTGCACGGT TATCAAGTGG AGAACTTGCC ACAATACAGC
201 CTTGGTTATG TTTATTTACC TGCATTGTTA GCGATTGCTA CAACATCGAT
251 GCTTACCACG CGAATTGGCG CTAAACTTGC CACCCAAATG CCAACAGCAA
301 GGCTTAAGCG ATTCTTTGCC ATTTTTTTAA TGTGCGTCGC TGTGACCATG
351 TTGTTCCAGT AATACTCATT GTTTATAGAG AAGGTTTGTT ATGCCTCAGG
401 GTTATCTGCA GTTTCCCAAT ATTGACCCCG TATTGTTTTC GATCGGCCCT
451 CTAGCGGTGC GCCTGTCTCT TATACACATC TCAACCCTTA CCGGTAAGCT
501 TGCATGCCTG CAGGTCGACT CTAGAGGATC CCCGCACGGT TGATGAGAGC
551 TGAAGACTGA AGTTCCTATT CTCTAGAAAG TATAGGAACT TCTGATCGAT
601 GATGGTTGAG ATGTGTATAA GAGACAGCTG TCGCGGTTTG TATCAAGACC
651 GTGTAGCAGC AAAATAGGGT AGTTAGGTGA AACAGTATTT AGATCTTTGT
701 CAGCGCATCG TCGATCAAGG TGTTTGGGTT GAAAATGAAC GAACGGGCAA
751 GCGTTGTTTG ACTGTGATTA ATGCCGATTT GACCTACGAT GTGGGCAACA
801 ATCAGTTTCC TCTAGTGACT ACACGCAAGA GTTTTTGGAA AGCTGCCGTA
851 GCCGAGTTGC TCGGCTATAT TCGTGGTTAC GATAATGCGG CGGATTTTCG
901 CCAATTAGGT ACCAAAACCT GGGATGCTAA TGCCAATTTA AACCAAGCAT
951 GGCTCAACAA TCCTTACCGT AAAGGTGAGG ATGACATGGG ACGCGTGTAT
1001 GGTGTTCAGG GTAGAGCTTG GGCTAAGCCT GATGGTGGTC ATATTGACCA
1051 GTTGAAAAAG ATTGTTGATG ATTTGAGCCG TGGCGTTGAT GACCGAGGTG
1101 AAATTCTTAA CTTCTACAAT CCGGGTGAAT TTCACATGGG GTGTTTGCGC
1151 CCTTGCATGT ACAGCCATCA TTTTTCATTG CTGGGGGATA CCTTGTATCT
1201CAGTACT CAGCGTTCAT GTGATGTGCC CTTGGGGTTG AATTTCAACA
1251TGCAGGT TTATGTGTTC CTTGCGCTGA TGG

Section B. PCR assay for confirmation/identification of *V. cholerae* strains MS1955, MS1987 and MS1692

Method description (SOP)

1. **Purpose:**

To confirm the identity of individual *Vibrio cholerae* strains based on characteristic features in their genomes that can be confirmed by the polymerase chain reaction (PCR) using specific oligonucleotide primers.

The SOP described below was designed for the screening of master and consistency lots of the *Vibrio cholerae* strains used in the production of the Duochol oral cholera vaccine (OCV)

Principle of the method. Duochol contains two formalin-inactivated strains of El Tor O1 *V. cholerae* that are isogenic except for the presence or absence of the *wbeT* gene that determines the serotype. The strain with the intact *wbeT* gene (MS1987) has the Ogawa serotype whereas the strain without it (MS1955) has the Inaba serotype. Furthermore, the two strains carry a deletion of the entire region encoding the cholera toxin genes *ctxAB*. PCR assays using oligonucleotide primers that amplify the specific region of the genome encoding the *wbeT* gene will lead to amplicons of different characteristic sizes in the different strains that can easily be identified using agarose gel electrophoresis. A further PCR can be used to confirm the characteristic deletion of the chromosomal region that normally carries the ctxAB genes in both strains.

The vaccine also contains recombinant cholera toxin B subunit (CTB) that is produced by a strain of classical O1 *V. cholerae* (MS1692) that is engineered in a way that can also be easily identified using PCR assays. In this case the *ctxA* gene encoding the toxic component of cholera toxin has been deleted. Further, the strain maintains the plasmid by complementation of the essential *lgt* gene which has been deleted. With the appropriate primers, both chromosomal lesions lead to PCR amplicons that are significantly different from those generated from strains carrying the wild-type genes and can be used to confirm the identity of the strain.

2. **Safety Precautions:**
   - **2.1.** The responsible scientist (or her approved designee) is responsible for ensuring that any staff appointed to perform the assay(s) has read and understood this SOP prior to performing the procedures described.

2.2. All work areas and equipment must be decontaminated with 70% ethanol at the end of the procedure.

2.3. Place all tips into a sharps container and place all materials that have encounter biological agents into the biological waste bags.

2.4. All biohazard waste bags, and sharps containers must be autoclaved according to DDR_SOP0010 (see Reference 15.1).

2.5. MSDS (Material Safety Data Sheet) of chemicals used in the procedures should be reviewed prior to working to ensure appropriate PPE (Personal Protective Equipment) and procedures are followed

3. Equipment:

Single channel pipette	Thermo Scientific
1.5ml Microcentrifuge tubes	Sartorius
0.2ml Microcentrifuge tubes	Sartorius
Microcentrifuge	Eppendorf 5415D
Heating block (95°C)	VWR Scientific
PCR machine	Eppendorff
Mupid-One agarose gel Electrophoresis unit	Takara
Imaging system e.g. Gel Doc EZ system.	Biorad

4. Reagents:

**Reagent**	**Manufacturer**	**Cat. No**
V. cholerae O1 El Tor Inaba	Engineered strain	Strain MS1955
V. cholerae O1 El Tor Ogawa	Engineered strain	Strain MS1987
V. cholerae O1 classical Inaba	Engineered strain	Strain MS1692
V. cholerae O1 El Tor Inaba	Wild-type strain	Phil6973
V. cholerae O1 classical Inaba	Engineered strain	JS1569(CVD103)
V. cholerae O1 classical Inaba	Wild-type strain	569B
Luria Bertani (LB) broth	Thermo Fisher	12780052
LB Agar	Thermo Fisher	22700025
2xPCR BIO HS Taq Mix Red	PCR biosystems	PB10.23.02
PCR primers	Eurofins	See appendix 1, tables I and II
Agarose	Fisher Scientific	BP1356-500
TAE buffer (x50)	Fisher Scientific	BP1332-1
GeneRuler DNA ladder mix	Thermo Fisher	SM0334
Midori green Direct Fluorescent DNA stain	Lubio Science	MG04

5. **Buffer and Reagent Preparations for tests:**

TAE 0.5x To prepare the buffer dilute the x50 TAE stock 1:100 (10ml of the concentrate plus 900ml deionized water. The composition of the TAE buffer is 40 mM Tris, 20 mM acetic acid, and 0.4 mM EDTA.)

6. **Before the experiment:**

6.1. Always ensure that all equipment used (e.g., micropipettes, spectrophotometer, microplate reader, etc.) has been properly calibrated and maintained.

6.2. Document all relevant information in the assay record sheet: Date, person doing the assay, person approving the results, type of material being tested etc.

7. **Method:**

7.1. Brief summary of the assay procedure Following the revival of strains from their respective seed lots, they are grown in liquid culture overnight. Cells from the culture are harvested by centrifugation and resuspended in water. Template DNA is prepared by boiling the cell suspension in a water-bath (or heating in a heating block) for 5-10 minutes. Cell debris is then removed by centrifugation. Individual PCR reactions on the template DNA using primer pairs designed to amplify the chromosomal regions that have been altered are done using a commercially available reaction master mix. The resulting amplified DNA is analyzed by agarose gel electrophoresis in which generated fragments are compared with a DNA size standard and controls generated from DNA of wild type strains.

7.2. DNA template preparation:

7.2.1. Take out a frozen vial of strain MS1955 (El Tor, Inaba) or MS1987(El Tor, Ogawa) from -70 ^{0}C.

7.2.2. Inoculate 2% of inoculum into LB broth (10 ml in 50 ml falcon tube). Do not refreeze the bacterial stock and discard after streaking out a sample on an LB agar plate (see below). Culture bacteria at 37° C overnight in shaking at 150-180 RPM.

7.2.3. From the same vial take a loopful of the cell suspension and streak out to single colonies on an LB agar plate. Incubate overnight at 37° C.

7.2.4. Next day, check the plate and make sure that the colonies are uniform in size and appearance.

7.2.5. Transfer 1.5 ml from the overnight liquid culture into a 1.5 ml centrifuge tube and spin down at 17,000xg for three minutes.

7.2.6. Carefully remove the supernatant from the tube, removing as much liquid as possible.

7.2.7. Add 150 µl of sterile distilled water to the tube and vortex to resuspend the cells

7.2.8. Place the tube in a heating block at 98°C and incubate for seven minutes.

7.2.9. Immediately transfer the tubes to a microcentrifuge and spin at 17,000 x g for five minutes.

7.2.10. Transfer the supernatant to a fresh sterile microcentrifuge tube and discard the tube containing the cell pellet.

7.3. Primer preparation:

7.3.1. Primers for the different PCR reactions are ordered from Eurofins Genomics. The oligonucleotides come as lyophilized powder. The amount they need to be dissolved in to achieve a stock concentration of 100 pmoles/µl is given in the Oligonucleotide Synthesis Report.

7.3.2. Each oligo is dissolved in sterile deionized water to the stock concentration of 100 pmoles/µl. Add the appropriate amount of water and vortex briefly. The dissolved primers can be stored at 4^0C.

7.3.3. Primers are added into primer mixes in pairs according to the protocol shown in S2.

7.3.4. To prepare primer mixes place 1µl of each stock solution of the primer pair to be tested into a sterile microcentrifuge tube. Add 8µl of sterile deionized water and mix. Spin briefly to bring the entire mixture to the bottom of the tube. The final concentration of each primer in the mixture is 10 pmoles/µl. If many samples are to be assayed using the same primer mix increase the volume prepared by increasing the volumes of each reagent proportionately.

7.4. PCR reactions:

7.4.1. PCR reactions are done in 25µl. To a sterile 0.2ml a microcentrifuge tube add:

DNA template (as prepared in sections 4.2)	5µl
Appropriate primer mix (as prepared in sections 4.3)	1.5µl
dH_2O	6.0µl
X2 PCR master mix	12.5µl

7.4.2. Mix the tube contents by vortexing briefly and spin briefly to bring the entire mixture to the bottom of the tube.

7.4.3. Place the tubes in the PCR machine and run the appropriate program as detailed in appendix 2.

7.4.4. **Controls:** Negative controls should be run from time to time to confirm specificity and determine whether the template DNA contributes any background. These should include reagent blanks with primers but no amplifiable DNA, reactions with DNA but no primers and full reactions mixtures including primers and DNA that are not subjected to amplification.

7.5. Analysis of the Amplified products:

7.5.1. 1% agarose gels are run in 0.5xTAE buffer. To prepare the buffer dilute the x50 TAE stock 1:100 (10ml of the concentrate plus 900ml deionized water).

7.5.2. Take 150ml of the diluted buffer (0.5xTAE) in a 250ml bottle and add 1.5g agarose. Heat in a microwave oven until the agarose dissolves completely giving a 1% agarose gel solution.

7.5.3. Transfer the amount of gel required to another bottle or flask. Allow the gel to cool to around 45°C. Add 1µl of the Midori Green Advance DNA stain per 25ml of gel. Pour gels appropriate for the number of samples using the gel holders, pouring frame and well combs supplied with the Mupid-One electrophoresis unit. The small gels can run eight samples, the large gels can run 17 samples. Allow the gel(s) to cool to room temperature before transferring to the electrophoresis unit.

7.5.4. When the gels have set remove the well comb and transfer to the Mupid-One electrophoresis unit. Add x0.5xTAE to the reservoir such that the gels are completely submerged.

7.5.5. Both the size standard (GeneRuler DNA ladder mix) and the x2 PCR master mix contain running dye and can be added directly to the gel. Load 2-3 μl samples of the amplified DNA and 4-5 l of the GeneRuler DNA ladder mix. The amplified samples can be diluted to 5 l with deionized water to facilitate handling.

7.5.6. Run the samples at 100V for 30 minutes. Visualize under UV light and take a photograph using an appropriate gel imaging system.

8. Data analysis and calculation

8.1. From the captured images of the gels confirm the presence or absence of bands of the appropriate sizes by reference to the size standards. Confirm that the sizes conform to the predicted sizes according to the samples amplified.

9. Quality control and acceptance criteria

9.1. The results are accepted when the amplified samples and controls give clearly amplified bands with the predicted sizes or no amplification at all if this is the predicted outcome as described in Appendix 1.

9.2. Control amplifications run without DNA or without primers should give no bands and controls containing all the reagents including primers and DNA but no subjected to amplification should also give no bands.

- **Primers for PCR analysis:**

Table S3 Primers for analysis of strains MS1955, MS1987 and Phil6973

Target gene	Primer pair	Sequence (5◎→3◎)
wbeT	wbeT F<br>wbeT R	GGTCAACAATGCCCTTTCAGGTCCTCAAACC<br>GAACAGGAATTCACAGCACATCGCTATGCAC
ctxAB	ctxAB F<br>ctxAB R	TTTTCTGTTAAACAAAGGGAGCATTATATGGTAAAG<br>CGGTTGCTTCTCATCATCGAACCAC
CTXf region	CTXF F<br>CTXF R	CCTCTATACTGCGACAGCGGCAGGTGAG<br>GTGGAACTCGGAGCAGGTAATGACTTTG

Table S4 Expected fragment sizes:

Gene	Strain(s)	Size (bp)
wbeT	MS1955	278

	MS1987<br>Phil6973	1067<br>1067*
ctxAB	MS1955<br>MS1987<br>Phil6973	No amplification<br>No amplification<br>1233
CTXf region	MS1955<br>MS1987<br>Phil6973	2065<br>2065<br>**

*Phil 6973 is Inaba, but the phenotype is due to a point mutation introducing a stop codon and using PCR the product is indistinguishable from an Ogawa strain such as MS1987.

** The wild type strain contains multiple copies of the CTXf region and can give very large amplicons (>10Kb). Under the PCR reaction conditions used no bands are expected. This is not the case for the either MS1955 or 1987 in which the entire region has been deleted.

Table S5 Primers for analysis of strains MS1692, CVD103(JS1569) and 569B

Target gene	Primer pair	Sequence (5@3@)
ctxAB	ctxAB F<br>ctxAB R	TTTTCTGTTAAACAAAGGGAGCATTATATGGTAAAG<br>CGGTTGCTTCTCATCATCGAACCAC
Lgt	lgt F<br>lgtR	GTTTTGCGATTGCGATATCGGGCATGATTGG<br>GGCTGTACATGCAAGGGCGCAAACACC

Table S6 Expected fragment sizes:

Gene	Strain(s)	Size (bp)
ctxAB	MS1955<br>JS1569<br>569B	663<br>663<br>1233
lgt	MS1692<br>JS1569<br>569B	918<br>1585<br>1585

Table S7 PCR amplification conditions:

Temperature (° C)	Time	Cycles
98	30 s	X1
98	15 s	X30
60	15 s	
72	1 m 30s	
72	5 m	X1
10	∞	Hold

The same conditions can be used for all amplifications.

Section C. Potential for co-culture of isogenic Inaba and Ogawa vaccine strains

Grown separately in both shake flask cultures and fermenter, the isogenic Duochol Inaba (MS1955) and Ogawa (MS1987) vaccine strains display identical growth curves as illustrated in Figure 3D in the main article. As shown in the experiments described below the isogenic Inaba and Ogawa strains can also be co-cultured together with equal multiplication of both strains.

Methods

Bacteria were revived from -80°C glycerol stock on LB-Agar plates at 37°C for 16h. 3 colonies were used for inoculation in 5 ml LB medium for 4h in 37°C at 180 rpm. Strains were set to the same OD by diluting with PBS.

The growth of the isogenic strains in competition experiments was tested using strains carrying plasmids pML-GreenFPλ/cI857 (Inaba) and pML-BlueFPλ/cI857 (Ogawa) that express the green fluorescent protein and blue fluorescent protein respectively when induced by incubation at 42°C. The pML-GreenFPλ/cI$_{857}$ and pML-BlueFPλ/cI$_{857}$ plasmids were kindly provided by pML-Biokonsult AB (Sweden). The plasmids are essentially identical except for small differences in the structural genes of the fluorescent proteins, and the plasmids of 3.5 Kb differ in only 12 bases. Thus the overall effect of the plasmids on growth should be minimal and the same which was checked when culturing strains with and without plasmids side by side in LB medium. Furthermore, the fluorescent proteins are not expressed during the competition experiments since these were performed at 30°C.

Briefly, isogenic pairs of strains, one carrying the pML-GreenFPλ/cI857 plasmid and pML BlueFPλ/cI857 were grown up overnight in 5 ml LB broth supplemented with ampicillin (100 µg/ml). The cells were washed and resuspended in PBS and the OD600 was adjusted to 0.5. The cells were then mixed in different proportions, for the experiment described at a ratio of 1:1. 50 µl of the mixed cell suspension was used to inoculate 5 ml LB broth supplemented with ampicillin or M9 medium supplemented with ampicillin and different carbon sources. The resulting culture was incubated at 30°C for 14h with shaking (180 rpm).

Serial dilutions of the suspension were then spread onto LB agar plates supplemented with ampicillin (100 µg/ml) and incubated at 30°C overnight in order to determine the actual number of colony forming units (cfus). In order to determine the ratio of Inaba to Ogawa cells in the suspension after growth overnight at 30°C

the plates were transferred to 42°C and incubated for 4h in order to express the green and blue fluorescent proteins. The actual ratio of one serotype to the other was determined by counting the number of colonies expressing each of the fluorescent proteins.

In other experiments, the cultures were passaged every 14 hours over a period of five days and the ratio of Inaba to Ogawa cells determined.

Results

In order to check whether the two cell types compete with each other in mixed culture we introduced plasmids carrying fluorescent proteins with different colours as described. Results showed that the ratios remain stable both over 14 hours or 20 hours of culture and even with serially passaged cultures over no less than five days.

One set of such experiments, showing the ratio of Ogawa/Inaba in co-cultures over 20 or 24 hours in M9 medium with different carbon sources, is illustrated in the **Figures SA and SB** below. The Inaba and Ogawa bacteria were mixed in proportions aimed to be 50% of each and the mixtures were then cultured as indicated and the proportion of the two strains was estimated as indicated and wes found to remain stable within the error margin of the determinations.

A

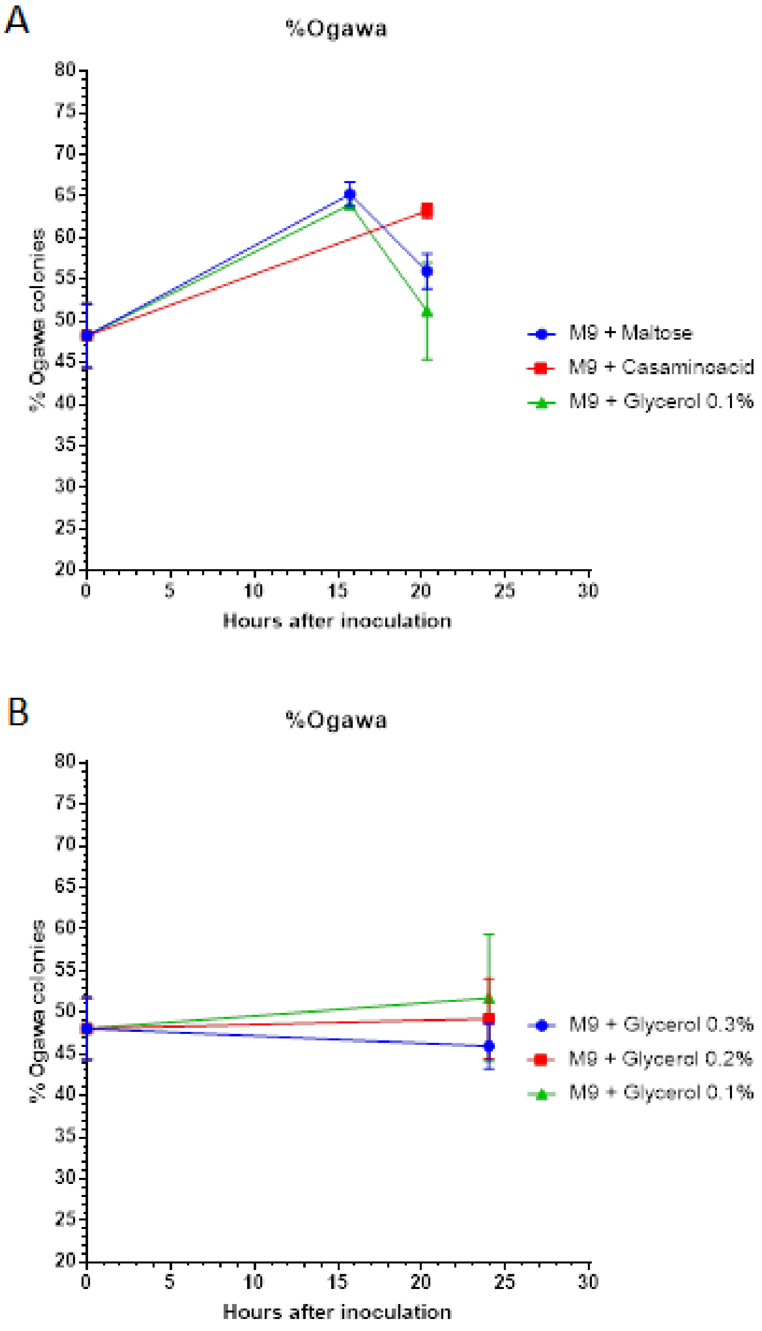

Figure SA&B: Ratio of Inaba to Ogawa in co-cultures grown in different M9 medium with different carbon sources A: Blue line; maltose, Red line; casamino acids, Green line 0.1% glycerol. B: Cultures were grown with different concentrations of glycerol as sole carbon and energy source. Blue line; 0.3% glycerol, Red line; 0.2% glycerol, Green line; 0.1% glycerol.

Section D- production of rCTB in 3 L fermentation

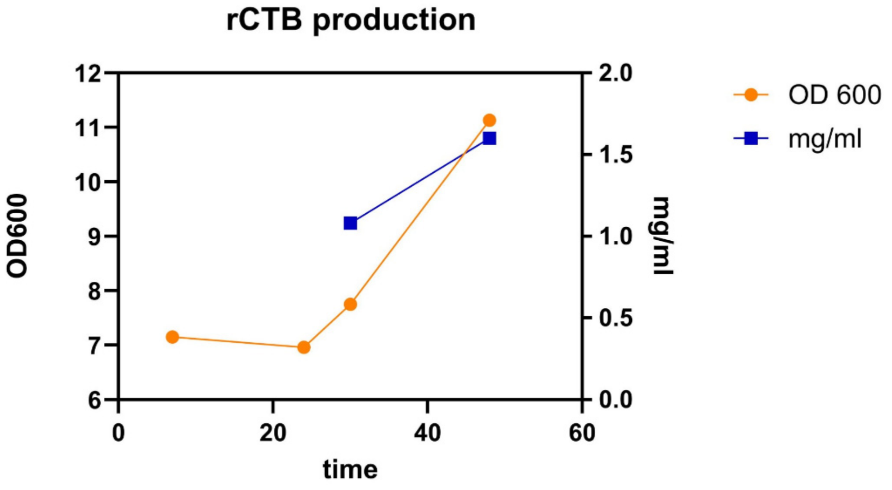

Figure SD. Growth curve (orange) of MMS1692 cell grown in 3L Fermenter. Amounts of rCTB (blue) at 24h and 48h test in GM1 Elisa.

Section E. Confirmation of stability at acid pH and rapid dissolution at neutral pH of Enprotect enteric capsule used for DuoChol OCV

The Capsugel® Enprotect® capsule used for the filling of the DuoChol OCV powder was chosen since it enables enteric delivery without the need for a post-filling coating step. This helps protect sensitive active pharmaceutical ingredients (APIs) during production and storage

As described by the Lonza manufacturer (https://media.ffycdn.net/eu/lonza-group-ag/zfvfYAtthEFSSxateCzG.pdf) Capsugel® Enprotect® capsule is completely stable for at 120 minutes in 0.1N HCl, and then when transferred to a buffer with pH 6.8 it completely dissolves within 20 minutes. Consistent enteric release to the jejunum/ileum region has been demonstrated, both with in vitro tests and trials in healthy volunteers, in both fasted and fed conditions.

We have as described below undertaken studies confirming the manufacturer´s in vitro pH study results:

1. **Studies of pH stability and release of Coomassie Blue dye from size 1 Enprotect capsules**

The photo **Figure** sequence below illustrates one of several similar tests undertaken to determine the capsule stability at low pH and kinetics of dissolution at neutral pH.

A size 1 Enprotect capsule from the same lot as used for preparing the DuoChol OCV prototype was opened, filled with Coomassie Blue powder, and closed in a similar manner as for filling a capsule with DuoChol. The capsule was then placed in a bottle with O.1N HCl which was incubated at room temperature for 120 minutes with slight agitation on a rotating drum. The flask was then emptied and instead filled with either phosphate buffered saline (PBS) with pH7.2 or as illustrated in the Figure with FaSSIF buffer with pH 6.8, and incubation continued until the capsule had been completely dissolved. FaSSIF (Fasted State Simulated Intestinal Fluid) is a biorelevant test medium that replicates small intestinal fluid in humans after drinking a glass of water; it was prepared as instructed by the manufacturer using components purchased from Biorelevant Ltd. (London, UK).

As seen in the **Figure SC** the capsule did not change in appearance, nor was there any release of blue dye into the medium during the whole 120 minutes of incubation

in HCl. After shifting the medium to FaSSIF or PBS one saw within minutes a progressive visual change in appearance of the capsule associated with release of dye into the medium. Dissolution of the capsule was complete after ca 20 minutes both in FaSSIF and in PBS.

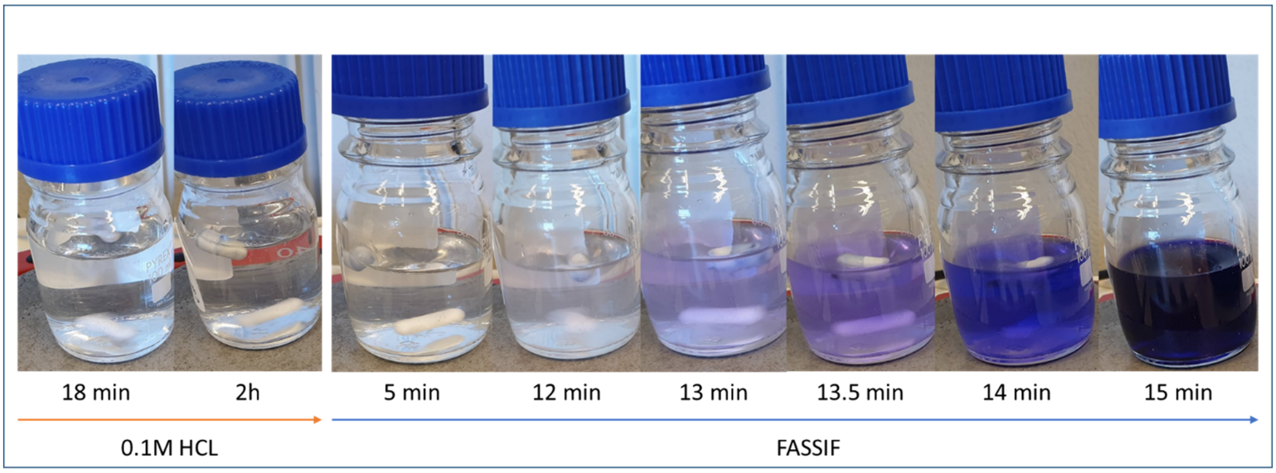

Figure SC. Dissolution test with FASSIF

2. Studies of pH stability and release of DuoChol OCV from size 1 Enprotect capsules

Similar studies were undertaken with three DuoChol OCV capsules, each filled with 150 mg lyophilized OCV powder and subjected to the same sequence of incubation, first for 120 minutes in 0.1N HCl and then in PBS until the capsules had dissolved completely. In addition to the visual inspection of the capsules over time, the recovery of LPS and CTB antigens was determined on samples from the PBS incubated/dissolved capsules using LPS Inhibition ELISA and GM1-ELISA, respectively, and freshly PBS dissolved capsule powder as reference.

Results showed, similar as for the Coomassie Blue filled capsules described above, that capsules were visually completely intact during the 120 minute incubation in HCl, and then rapidly and progressively dissolved in PBS within 30 minutes. Samples collected after 30 minutes in PBS were found to contain 68-100% of the expected antigen level, and samples analyzed after 55 minutes of capsule incubation in PBS contained 100% of the reference preparation antigen levels.

Original images from the main Manuscript

Figure 3

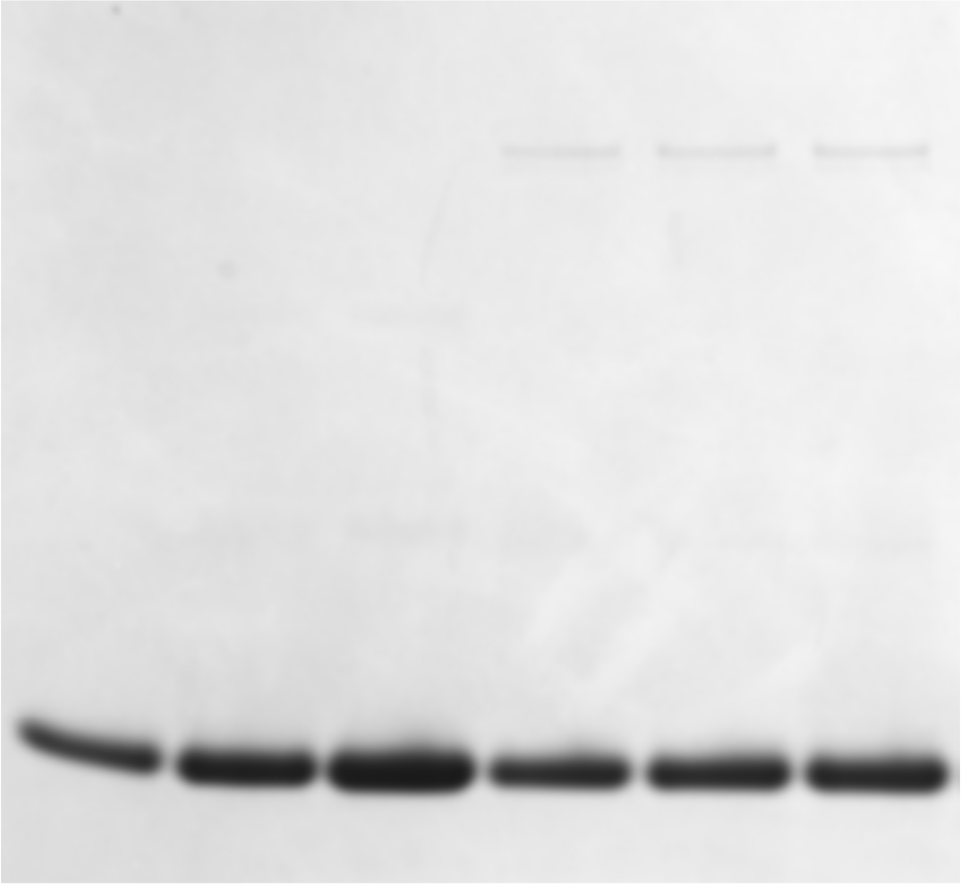

Figure 5

Time 6 months storage

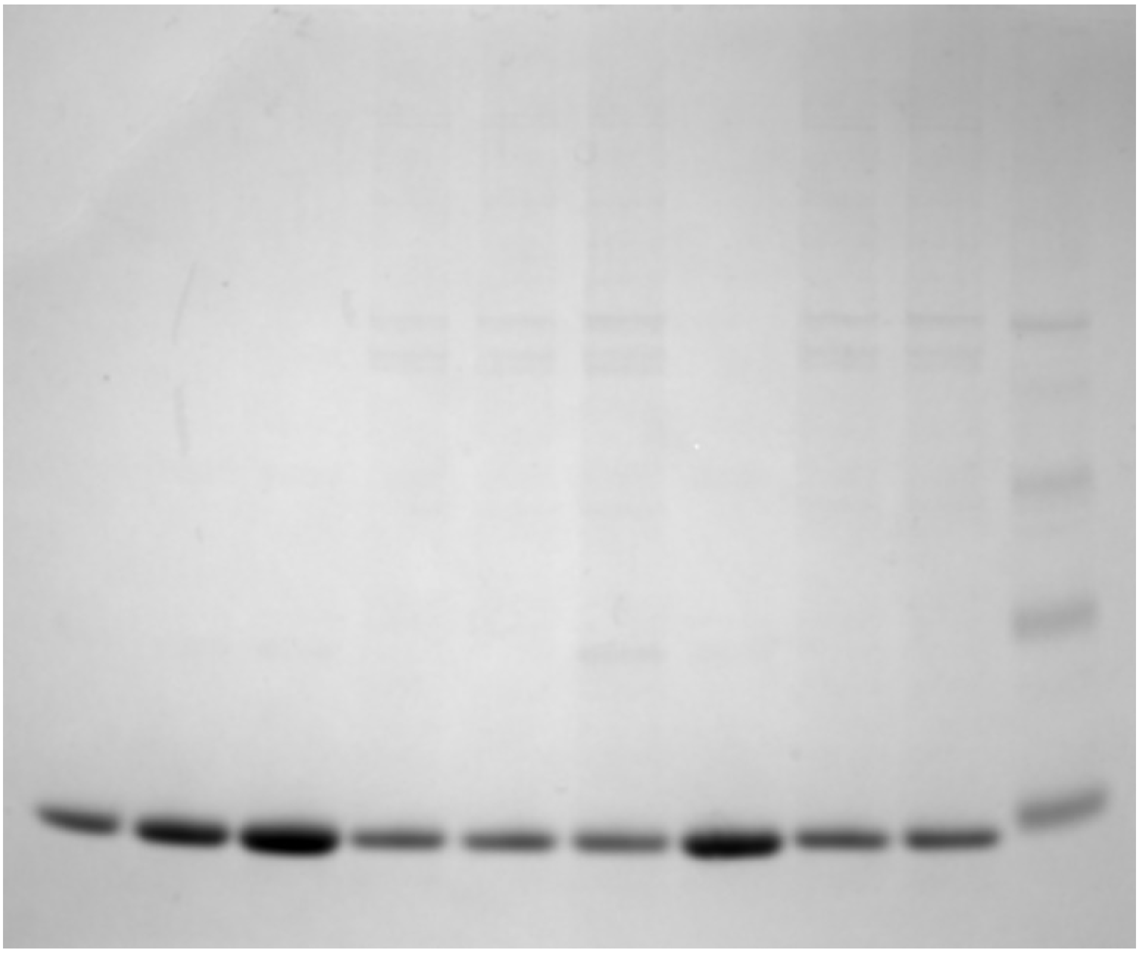

Figure 5

Time 21 months storage

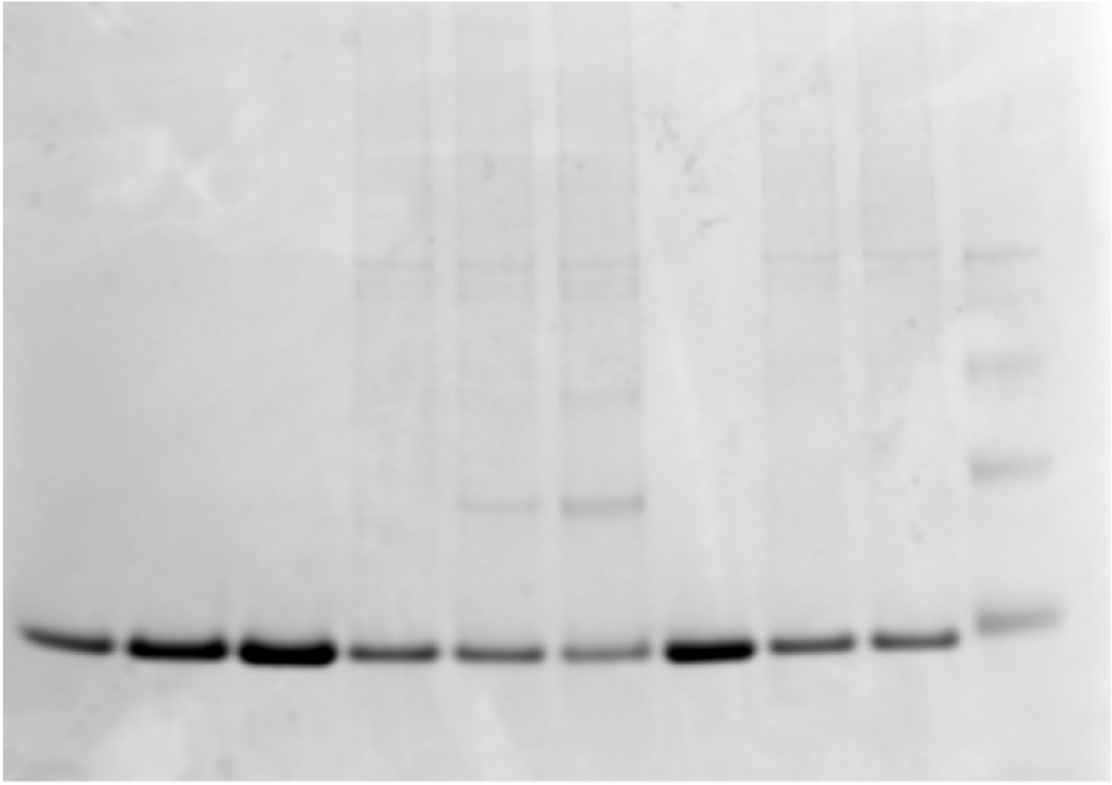

Supplement: Supplementary file 1 [file vaccines-14-00573-s001.zip › vaccines-4331617-supplementary/vaccines-4331617-supplementary.pdf]
